# High intensity exercise programme in patients with hypertrophic cardiomyopathy: a randomized trial

**DOI:** 10.1093/eurheartj/ehae919

**Published:** 2025-03-03

**Authors:** Joyee Basu, Dimitra Nikoletou, Chris Miles, Hamish MacLachlan, Gemma Parry-Williams, Fred Tilby-Jones, Paulo Bulleros, Zephryn Fanton, Claire Baker, Shane Purcell, Carmen Lech, Tracy Chapman, Peter Sage, Shams Wahid, Nabeel Sheikh, Shruti Jayakumar, Aneil Malhotra, Tracey Keteepe-Arachi, Belinda Gray, Gherardo Finocchiaro, Gerald Carr-White, Elijah Behr, Maite Tome, Jamie O’Driscoll, Irina Chis Ster, Sanjay Sharma, Michael Papadakis

**Affiliations:** Cardiovascular and Genomic Research Institute, City St George’s, University of London, Cranmer Terrace, London SW17 0RE, UK†; Cardiovascular Clinical Academic Group, St George’s University Hospitals NHS Foundation Trust, Blackshaw Road, London SW17 0QT, UK†; Cardiovascular and Genomic Research Institute, City St George’s, University of London, Cranmer Terrace, London SW17 0RE, UK†; Cardiovascular and Genomic Research Institute, City St George’s, University of London, Cranmer Terrace, London SW17 0RE, UK†; Cardiovascular Clinical Academic Group, St George’s University Hospitals NHS Foundation Trust, Blackshaw Road, London SW17 0QT, UK†; Cardiovascular and Genomic Research Institute, City St George’s, University of London, Cranmer Terrace, London SW17 0RE, UK†; Cardiovascular Clinical Academic Group, St George’s University Hospitals NHS Foundation Trust, Blackshaw Road, London SW17 0QT, UK†; Cardiovascular and Genomic Research Institute, City St George’s, University of London, Cranmer Terrace, London SW17 0RE, UK†; Cardiovascular Clinical Academic Group, St George’s University Hospitals NHS Foundation Trust, Blackshaw Road, London SW17 0QT, UK†; Cardiovascular and Genomic Research Institute, City St George’s, University of London, Cranmer Terrace, London SW17 0RE, UK†; Cardiovascular and Genomic Research Institute, City St George’s, University of London, Cranmer Terrace, London SW17 0RE, UK†; Cardiovascular Clinical Academic Group, St George’s University Hospitals NHS Foundation Trust, Blackshaw Road, London SW17 0QT, UK†; Cardiovascular and Genomic Research Institute, City St George’s, University of London, Cranmer Terrace, London SW17 0RE, UK†; Cardiovascular Clinical Academic Group, St George’s University Hospitals NHS Foundation Trust, Blackshaw Road, London SW17 0QT, UK†; Cardiovascular and Genomic Research Institute, City St George’s, University of London, Cranmer Terrace, London SW17 0RE, UK†; Cardiovascular and Genomic Research Institute, City St George’s, University of London, Cranmer Terrace, London SW17 0RE, UK†; Cardiovascular Clinical Academic Group, St George’s University Hospitals NHS Foundation Trust, Blackshaw Road, London SW17 0QT, UK†; Cardiovascular and Genomic Research Institute, City St George’s, University of London, Cranmer Terrace, London SW17 0RE, UK†; Cardiovascular Clinical Academic Group, St George’s University Hospitals NHS Foundation Trust, Blackshaw Road, London SW17 0QT, UK†; Cardiovascular and Genomic Research Institute, City St George’s, University of London, Cranmer Terrace, London SW17 0RE, UK†; Cardiovascular Clinical Academic Group, St George’s University Hospitals NHS Foundation Trust, Blackshaw Road, London SW17 0QT, UK†; Cardiovascular and Genomic Research Institute, City St George’s, University of London, Cranmer Terrace, London SW17 0RE, UK†; Cardiovascular Clinical Academic Group, St George’s University Hospitals NHS Foundation Trust, Blackshaw Road, London SW17 0QT, UK†; Cardiovascular and Genomic Research Institute, City St George’s, University of London, Cranmer Terrace, London SW17 0RE, UK†; Cardiovascular Clinical Academic Group, St George’s University Hospitals NHS Foundation Trust, Blackshaw Road, London SW17 0QT, UK†; Guy’s and St Thomas’ NHS Foundation Trust, London, UK; Cardiovascular and Genomic Research Institute, City St George’s, University of London, Cranmer Terrace, London SW17 0RE, UK†; Cardiovascular and Genomic Research Institute, City St George’s, University of London, Cranmer Terrace, London SW17 0RE, UK†; Cardiovascular Clinical Academic Group, St George’s University Hospitals NHS Foundation Trust, Blackshaw Road, London SW17 0QT, UK; University of Manchester, Manchester, UK; Manchester Institute of Health and Performance, Manchester, UK; Cardiovascular and Genomic Research Institute, City St George’s, University of London, Cranmer Terrace, London SW17 0RE, UK†; Cardiovascular Clinical Academic Group, St George’s University Hospitals NHS Foundation Trust, Blackshaw Road, London SW17 0QT, UK†; Cardiovascular and Genomic Research Institute, City St George’s, University of London, Cranmer Terrace, London SW17 0RE, UK†; Cardiovascular Clinical Academic Group, St George’s University Hospitals NHS Foundation Trust, Blackshaw Road, London SW17 0QT, UK†; Cardiovascular and Genomic Research Institute, City St George’s, University of London, Cranmer Terrace, London SW17 0RE, UK†; Cardiovascular Clinical Academic Group, St George’s University Hospitals NHS Foundation Trust, Blackshaw Road, London SW17 0QT, UK†; Guy’s and St Thomas’ NHS Foundation Trust, London, UK; Cardiovascular and Genomic Research Institute, City St George’s, University of London, Cranmer Terrace, London SW17 0RE, UK†; Cardiovascular Clinical Academic Group, St George’s University Hospitals NHS Foundation Trust, Blackshaw Road, London SW17 0QT, UK†; Cardiovascular and Genomic Research Institute, City St George’s, University of London, Cranmer Terrace, London SW17 0RE, UK†; Cardiovascular Clinical Academic Group, St George’s University Hospitals NHS Foundation Trust, Blackshaw Road, London SW17 0QT, UK†; Cardiovascular Clinical Academic Group, St George’s University Hospitals NHS Foundation Trust, Blackshaw Road, London SW17 0QT, UK; Diabetes Research Centre, College of Life Sciences, University of Leicester, Leicester, UK; Cardiovascular and Genomic Research Institute, City St George’s, University of London, Cranmer Terrace, London SW17 0RE, UK†; Cardiovascular and Genomic Research Institute, City St George’s, University of London, Cranmer Terrace, London SW17 0RE, UK†; Cardiovascular Clinical Academic Group, St George’s University Hospitals NHS Foundation Trust, Blackshaw Road, London SW17 0QT, UK†; Cardiovascular and Genomic Research Institute, City St George’s, University of London, Cranmer Terrace, London SW17 0RE, UK†; Cardiovascular Clinical Academic Group, St George’s University Hospitals NHS Foundation Trust, Blackshaw Road, London SW17 0QT, UK†

**Keywords:** Hypertrophic cardiomyopathy, Cardiac rehabilitation, High intensity exercise, Sports cardiology

## Abstract

**Background and Aims:**

The feasibility and impact of high intensity exercise programmes in patients with hypertrophic cardiomyopathy (HCM) are unknown. This study was conducted to determine the feasibility of a high intensity exercise programme and explore safety and efficacy outcomes in patients with HCM.

**Methods:**

Participants were randomized to a 12-week supervised exercise programme (*n* = 40) in addition to usual care, or usual care alone (*n* = 40). All participants underwent assessment at baseline and 12 weeks. The exercise group was re-evaluated 6 months post-programme. Feasibility was assessed by (i) recruitment, adherence, and retention rates; (ii) staffing ratios; (iii) logistics; and (iv) acceptability of the intervention. The primary exploratory safety outcome was a composite of arrhythmia-related events. Exploratory secondary outcomes included changes in (i) cardiorespiratory fitness; (ii) cardiovascular risk factors; and (iii) quality of life, anxiety, and depression scores.

**Results:**

Overall, 67 (84%) participants completed the study (*n* = 34 and *n* = 33 in the exercise and usual care groups, respectively). Reasons for non-adherence included travel, work, and family commitments. Resource provision complied with national cardiac rehabilitation standards. There was no difference between groups for the exploratory safety outcome (*P* = .99). At 12 weeks, the exercise group had a greater increase in peak oxygen consumption (VO_2_) [+4.1 mL/kg/min, 95% confidence interval (CI) 1.1, 7.1] and VO_2_ at anaerobic threshold (+2.3 mL/kg/min, 95% CI 0.4, 4.1), lower systolic blood pressure (−7.3 mmHg, 95% CI −11.7, −2.8) and body mass index (−0.8 kg/m^2^, 95% CI −1.1, −0.4), and greater improvement in hospital anxiety (−3, 95% CI −4.3, −1.7) and depression (−1.7, 95% CI −2.9, −0.5) scores, compared to the usual care group. Most exercise gains dissipated at 6 months.

**Conclusions:**

A high intensity exercise programme is feasible in patients with HCM, with apparent cardiovascular and psychological benefits, and no increase in arrhythmias. A large-scale study is required to substantiate findings and assess long-term safety of high intensity exercise in HCM.

## Structured Graphical Abstract

**structured graphical abstract:**
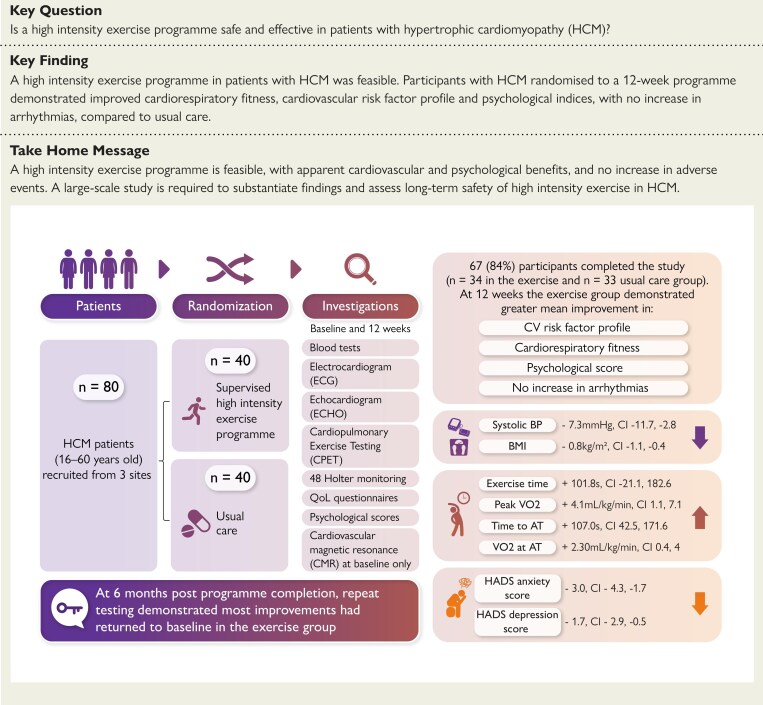
Change in cardiovascular risk factor profile, cardiorespiratory fitness, psychological scores, and occurrence of arrhythmias in individuals with HCM following participation in a 12-week supervised high intensity programme. AT, anaerobic threshold; BP, blood pressure; BMI, body mass index; CV, cardiovascular; HADS, hospital anxiety and depression scale.

**See the editorial comment for this article ‘Exercise prescription in hypertrophic cardiomyopathy: Dr Lown’s lesson to break taboos’, by I. Olivotto and F. D'Ascenzi, https://doi.org/10.1093/eurheartj/ehae659.**

## Introduction

Hypertrophic cardiomyopathy (HCM) is the most common inherited cardiac condition encountered in clinical practice^1^ and until recently was considered a leading cause of exercise-related sudden cardiac death (SCD) in young individuals.^2^ Historical exercise recommendations adopted a conservative stance, confining affected individuals to low intensity exercise regimes^3,4^ promoting sedentary behaviour.^5–7^ Presently, 55% of patients with HCM fail to meet the minimum physical activity (PA) recommendations.^5^ A sedentary lifestyle cultivates obesity,^6^ increases cardiovascular risk,^7–9^ and has considerable adverse psychological impact, particularly in younger individuals.^5,10–12^

Contemporary studies suggest that the risk of SCD during exercise in individuals with HCM may not be as high as initially perceived. Indeed, many highly active individuals and athletes are diagnosed incidentally after years of exercise, rather than due to symptoms or adverse events.^13^ Exercise programmes in individuals within the sixth and seventh decades of life suggest that moderate intensity exercise has a favourable effect on functional capacity and improves quality of life (QoL) with no signal for increase in major adverse events.^14–16^ A large population study of self-reported participation in moderate to vigorous intensity exercise in middle-aged individuals with HCM demonstrated reduced all-cause and cardiovascular mortality in those that exercised most.^17^ Most recently, a prospective study of 1534 individuals with HCM, self-reporting engagement in either vigorous, moderate, or sedentary exercise, demonstrated no difference in the occurrence of cardiac events between groups.^18^ Furthermore, small follow-up studies in athletes who continue to compete despite a diagnosis of HCM have not revealed an increase in adverse outcomes, or negative impact on cardiac phenotype.^19,20^ However, safety and outcome data from large-scale randomized trials, are lacking.

The shifting opinion is reflected in the most recent European Society of Cardiology (ESC) and American Heart Association (AHA) guidelines which support a more liberal approach towards exercise in individuals with HCM.^21,22^ In order to support the growing number of individuals with HCM wishing to exercise at higher intensities, there is a need to educate health professionals on exercise prescription, and to explore how to integrate high intensity exercise into disease-specific cardiac rehabilitation programmes. Therefore, we aimed to examine the feasibility of a supervised, individually tailored high intensity exercise programme in young and middle-aged individuals with HCM, and to explore safety and the effect on cardiorespiratory fitness, risk factors for atherosclerosis, and psychological and QoL parameters.

## Methods

### Study design

This was a multi-site randomized controlled feasibility study. Study participants were recruited between January 2018 and May 2019 at three tertiary centres in London, UK. Individuals were block randomized to a high intensity exercise programme in addition to usual care, or usual care alone. The study protocol is available in Supplementary data online, *Supplement S1*. All data analyses were blinded. Ethical approval was granted by the Yorkshire, and the Humber Research Ethics Committee and was approved by site institutional review boards. Written consent was obtained from all subjects. This study is registered with ClinicalTrials.gov NCT05459467.

### Outcomes

The feasibility of the programme was assessed by means of (i) response to invitation to participate and reasons for refusal; (ii) adherence to the cardiac rehabilitation programme; (iii) staffing and resource assessment; and (iv) acceptability of the intervention and educational material (see Supplementary data online, *Supplement S2*).

The primary exploratory safety outcome was a composite of cardiovascular death, cardiac arrest, appropriate or inappropriate implantable cardioverter defibrillator (ICD) therapy, exercise-induced syncope, sustained ventricular tachycardia (VT), non-sustained VT (NSVT), and sustained atrial arrhythmias (≥30 s).

Secondary exploratory outcomes focused on cardiovascular health and wellbeing, and were assessed by changes in: (i) cardiopulmonary exercise testing (CPET) parameters [total exercise time (tMax), peak oxygen consumption (pVO_2_/kg), time to anaerobic threshold (tAT), VO_2_ at AT (VO_2_/kgAT), and VE/VCO_2_ slope]; (ii) PA levels; (iii) baseline characteristics [blood pressure (BP), body mass index (BMI)]; (iv) psychological and QoL scores [Hospital Anxiety and Depression Scale (HADS), World Health Organization Disability Assessment Schedule II (WHO-DAS II), 36-Item Short Form Survey (SF-36)]; (v) biochemical parameters [glycated haemoglobin (HbA1c) and lipid profile]; (vi) cardiac markers [high-sensitivity troponin, and N-terminal pro-B-type natriuretic peptide (NT-proBNP)]; (vii) echocardiographic parameters [left atrial (LA) volume, left ventricular end-diastolic dimension (LVEDD), left ventricular wall thickness (LVWT)], diastolic parameters (E/E′, E/A); and (viii) burden of premature ventricular complexes.

### Study participants

The inclusion and exclusion criteria were developed to prioritize safety and include individuals representative of real-world patients with HCM, who were able to participate in high intensity exercise. Participants were recruited sequentially from the inherited cardiac conditions clinics at each site. Eligible patients were those with a diagnosis of HCM (LVWT ≥ 15 mm in the absence of abnormal loading conditions), aged 16–60 years, New York Heart Association classes I–II, able to exercise, and commit to the protocol defined programme duration. Exclusion criteria included competitive athletes, history of exercise-induced syncope, poorly controlled ventricular arrhythmias, surgical myectomy, severely reduced left ventricular (LV) ejection fraction (<35%), LV outflow tract gradient ≥ 50 mmHg at rest, following Valsalva manoeuvre or squatting, awaiting or recent device implantation (<3 months), known coronary artery disease (CAD) (lesion > 50% on coronary angiography/previous coronary intervention), exercise limited by a non-cardiac cause, renal failure, phenocopies of HCM, and pregnancy. Patients continued their prescribed medical therapy.

### Study procedures

All study participants underwent baseline (T0) evaluation with detailed clinical and exercise history (see Supplementary data online, *Supplement S3*), physical examination, routine blood tests, 12-lead ECG, transthoracic echocardiogram, maximal CPET, 48 h ECG monitor, and cardiac magnetic resonance (CMR) imaging (see Supplementary data online, *Supplement S4*). Psychological and QoL assessment was performed using standardized questionnaires (HADS, WHO-DAS II, SF-36) (see Supplementary data online, *Supplement S5*). Individuals assigned to the exercise arm underwent consultation with a cardiac rehabilitation instructor. Participants with an ICD underwent interrogation of their device. All investigations, apart from CMR, were repeated in all study participants at 12 weeks (T12). Participants in the exercise arm were re-evaluated at 6 months (T6m) post-exercise programme completion, with repeat CPET, 48 h ECG monitor and psychological and QoL questionnaires.

The exercise group participated in supervised exercise sessions for a total of 1 h, twice weekly, together with an hour per week of home-based exercise, for 12 weeks. Each exercise class included up to 10 participants. Participants began exercising at high intensity at 70% of heart rate reserve (HRR), progressing by 5% increments to 85% of HRR (see Supplementary data online, *Supplement S6*). This was calculated using the Karvonen formula^23^ using the maximum heart rate (HR) derived from the baseline CPET. For participants with an ICD, the target HR was set at least 10 b.p.m. below the activation threshold of the device. The Borg rating of perceived exertion scale was used to monitor exertion during the programme.^24^ Participants were provided with Polar (M430) watches to ensure that the prescribed intensity of exercise was maintained. To ensure safety, continuous ECG monitoring was performed during exercise classes. Exercise classes consisted of warm up exercises, followed by a circuit of alternating aerobic and resistance exercises, ending with a cool down period. A minimum of 50% of the hour was dedicated to aerobic activities. To accommodate for differences in baseline fitness levels and exercise capacity, a colour-coded programme was used [red—level 1 (HRR 70%–75%), orange—level 2 (HRR 75%–80%), green—level 3 (HRR 80%–85%)], which allowed each participant to progress in a graded fashion. Each time the exercise level was increased, a 48 h ECG monitor was attached to the patient in order to assess for arrhythmias. A pre-recorded exercise programme was also available, enabling patients to perform additional exercise sessions at home (see Supplementary data online, *Supplement S7*). Exercises were also detailed in booklets provided to patients (see Supplementary data online, *Supplement S2*). Engagement in the exercise programme was assessed using patient diaries (see Supplementary data online, *Supplement S2*) and Polar watch downloads. An educational session followed every exercise session. Topics covered included: home-based exercise sessions, benefits of exercise, living with HCM, medications, diet, stress/anxiety management and mindfulness, self-management/self-efficacy, and living with an ICD. At 12 weeks, individuals were instructed to continue with the frequency and intensity of PA achieved at the end of the cardiac rehabilitation programme, using the home-based exercise programme, and were re-evaluated at T6m.

### Statistical analysis

All data were analysed by an individual blinded to the group allocation. All the collected variables, pre- and post-intervention, have been visually inspected using graphics and summarized by intervention group according to their nature; means, standard deviations, medians, interquartiles, and ranges for continuous variables and proportions for categorical/binary variables. Change in exercise capacity from baseline to 12 weeks is presented using box whisker plots containing raw median, interquartile range, and range data. Per-protocol analyses (including only participants who completed the 12-week trial) have been conducted using adequate statistical tests. Unpaired type analyses (*t*-tests or χ^2^ tests) have been used for post-intervention measurements and their respective changes from baseline comparisons between groups according to their distributional assumptions. One-to-one transformations such as logarithm have been applied as necessary to meet the tests’ assumptions requirements. Intention-to-treat analyses (including all participants who were randomized) have also been conducted using appropriate permutation tests (non-parametric setting) under the missing completely at random assumption.^25–27^ Both per-protocol and intention-to-treat analyses are presented for the secondary outcomes to ascertain the influence of assuming normal distribution or any missing data. As a feasibility rather than hypothesis testing study, these analyses have exploratory value only and, therefore, do not require adjustments for the probability of a type 1 error. Therefore, the parameters are reported as 95% confidence intervals (CIs) rather than *P*-values. Mixed models have been applied to a series of clinically relevant longitudinal measurements under the normality assumption for the intervention group only, who were followed up for 6 months after the planned trial finished. These data are presented graphically as changes in mean and CIs. The advantage of this method is that it considers all the complete observations under the missing at random assumption for the missing observations, which is not testable from the data at hand.^28^ Statistical significance was cautiously set at *P* < .05 for the assessment of the exploratory analyses. All tests were two-sided, and the 95% CIs are presented to reflect the exploratory nature of the study. All analyses and graphics have been conducted in STATA (StataCorp. 2023. Stata Statistical Software: Release 17. College Station, TX: StataCorp LLC).

## Results

**Figure 1:**
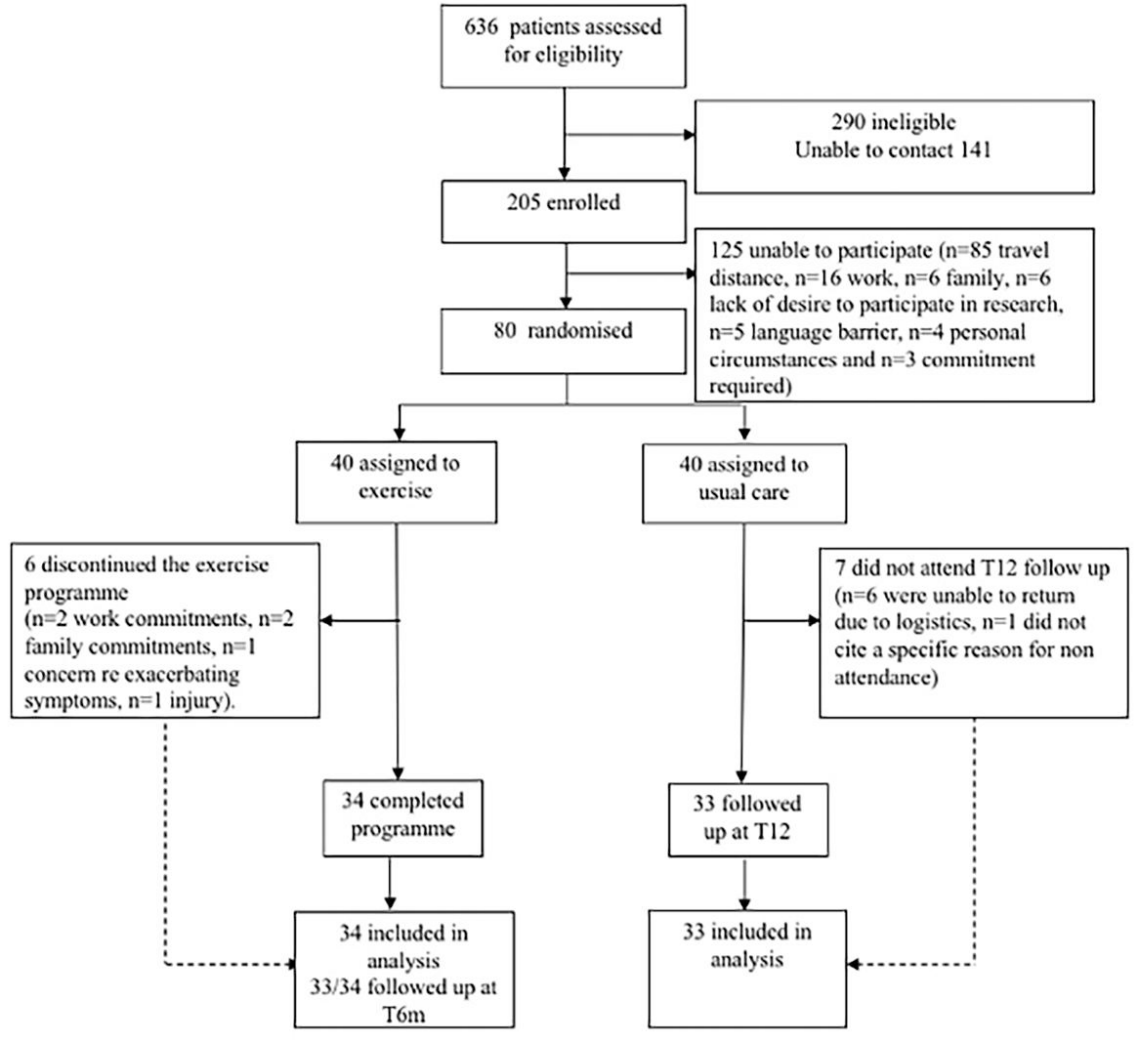
Recruitment and adherence flow chart

Between January 2018 and May 2019, 636 patients with HCM, aged between 16 and 60 years, were identified as potential participants, and screened for eligibility. Of the 205 individuals contacted, 80 agreed to participate. Commonly cited reasons for non-participation included travel distance (*n* = 85), work (*n* = 16) and family (*n* = 6) commitments, lack of desire to participate in research (*n* = 6), language barrier (*n* = 5), personal circumstances (*n* = 4), and commitment required (*n* = 3). Individuals were randomized to exercise (*n* = 34 completed the programme) or usual care groups (*n* = 33 completed follow-up at T12) (*Figure 1*).

Baseline characteristics are presented in (*Table 1*). There was no significant difference in baseline values, except for HbA1c which was higher in the exercise group. Of note, only two individuals in the exercise group and one individual in the usual care group were diabetic.

**Table 1:** Baseline characteristics

	Exercise	Usual care	*P*-value
	*n* = 40	*n* = 40	
Age, years	48 (7.9)	44 (8.9)	.053
PA per week, hours	3.0 (1.0–4.0)	4.0 (2.0–6.0)	.073
Sex			
Men	36 (90%)	31 (77.5%)	.225
Women	4 (10%)	9 (22.5%)	
Ethnicity			
White	22 (55%)	28 (70%)	.379
Black	11 (27.5%)	7 (17.5%)	
Other^a^	7 (17.5%)	5 (12.5%)	
Genetic testing			
Performed	29 (73%)	29 (73%)	1.000
Pathogenic variant identified	8 (28%)	8 (28%)	1.000
Medical history			
Hypertension	12 (30%)	14 (35%)	.812
Diabetes	2 (5%)	1 (2.5%)	1.000
Hypercholesterolaemia	14 (35%)	5 (12.5%)	.069
TIA	1 (2.5%)	0 (0%)	1.000
Renal failure	1 (2.5%)	1 (2.5%)	1.000
Family history			
SCD	6 (15%)	8 (20%)	.770
HCM	18 (45%)	17 (42.5%)	1.000
Primary prevention ICD	6 (15%)	3 (7.5%)	.481
Medical therapy			
BB	13 (32.5%)	9 (22.5%)	.453
Non-DHP CCB	3 (7.5%)	0 (0%)	.241
DHP CCB	6 (15%)	7 (17.5%)	1.000
Cardiovascular risk factors			
BMI, kg/m^2^	28.1 (25.5–30.3)	27.1 (24.8–31.6)	.641
HR, b.p.m.	62.5 (58.8–70.3)	60.0 (54–73.8)	
SBP, mmHg	122.0 (115.0–137.3)	120 (110.0–130.0)	.271
DBP, mmHg	80.0 (70.0–83.0)	80.0 (70.0–80.0)	.635
HbA1c, mmol/mol	37.0 (35.0–38.8)	36.0 (32.5–37.5)	.036
Cholesterol, mmol/L	4.8 (3.9–5.5)	4.9 (4.2–5.6)	.368
Cardiac biomarkers			
NT-proBNP, ng/L	248.5 (90.3–365.0)	239.0 (63.0–467.0)	.941
Troponin, ng/L	9.0 (6.0–16.0)	11.0 (5.0–20.0)	.735
CPET parameters			
Total exercise time (tMax), s	690 (592–774)	670 (564.5–761.3)	.495
Time to AT (tAT), s	459 (348.5–569.3)	415.5 (359–498.8)	.395
Peak VO_2_ (pVO_2_/kg), mL/kg/min	28.3 (23.0–34.9)	30.3 (21.7–35.2)	.917
VO_2_ at AT (VO_2_/kgAT), mL/kg/min	22.7 (17.7–28.6)	20.3 (15.3–26.9)	.371
VE/VCO_2_	28.0 (25.4–29.9)	27.5 (25.0–30.4)	.741
Echocardiographic parameters			
LVEF, %	65 (60–70)	65 (60–70)	.892
Maximum LVWT, mm	16.0 (14.0–18.8)	16.0 (15.0–18.0)	.984
LVEDD, mm	47.5 (44.0–51.0)	48.0 (44.0–51.0)	.943
LVOT gradient at rest, mmHg	4.5 (3.6–6.6)	5.0 (4.0–8.0)	.061
LVOT gradient on provocation, mmHg	6.8 (4.9–15)	12.0 (9.0–23)	.053
LA volume, mL	72.0 (53.0–95.0)	70.0 (57.0–96.5)	.947
E/A	1.3 (1.0–1.5)	1.3 (0.9–1.6)	.927
Average E/E′	7.5 (5.8–9.7)	7.4 (6.4–10.2)	.534
Ventricular ectopics (48 h)	16.0 (3.0–242.0)	6.0 (1–75.3)	.096
Psychological and QoL scores			
HADSD score	4.0 (.10–6.0)	2.0 (0.0–3.0)	.090
HADSA score	6.0 (2.0–9.0)	5.0 (3.0–6.0)	.229
WHO-DAS II score	2.0 (0.0–14.0)	2.0 (0.0–7.0)	.634
SF-36 score	65.0 (50.0–80.0)	70.0 (50.0–85.0)	.849

Data are presented as median (IQR) or *n* (%) unless otherwise stated.

AT, anaerobic threshold; BB, beta blocker; BMI, body mass index; CCB, calcium channel blocker; DHP, dihydropyridine; DBP, diastolic BP; HADSA score, Hospital Anxiety and Depression Scale anxiety score; HADSD score, Hospital Anxiety and Depression Scale depression score; HCM, hypertrophic cardiomyopathy; HR, heart rate; ICD, implantable cardioverter defibrillator; LA, left atrium; LVEDD, left ventricular end-diastolic dimension; LVWT, left ventricular wall thickness; non-DHP, non-dihydropyridine; NT-proBNP, N-terminal pro-B-type natriuretic peptide; PA, physical activity; QoL, quality of life; SBP, systolic BP; SCD, sudden cardiac death; SF-36, short form 36; TIA, transient ischaemic attack; VE/VCO_2_, minute ventilation/carbon dioxide production; WHO-DAS II, World Health Organization Disability Assessment Schedule II.

^a^Other comprised Asian or mixed race.

### Feasibility

The overall retention rate was 85% and 82.5% in the exercise and usual care groups, respectively. Thirty-four participants in the exercise group completed the exercise programme and 33 returned at 6 months for repeat evaluation. Of those who completed the exercise programme, all attended the minimum of 75% of sessions and 64.7% reached the maximum target HRR of 85%. Resources including staffing and equipment were in accordance with recommended standards as defined by the British Association for Cardiovascular Prevention and rehabilitation (BACPR) and the American Association of Cardiovascular and Pulmonary Rehabilitation (ACPICR).^29,30^

As a perceived higher risk population, a staffing ratio of 1:5, with additional onsite physician supervision, ensured that each session could be safely delivered. All staff were trained in basic life support, and individuals were monitored safely throughout by means of self-monitoring for adverse symptoms, watches, and continuous ECG monitoring. There was immediate access to cardiac resuscitation equipment and an emergency team as required. There was sufficient equipment to deliver each exercise session including stationary bicycles, treadmills, a mini trampoline, weights, and therabands. Participants were given the opportunity to provide feedback, which reflected that the intervention and associated educational materials were well received (see Supplementary data online, *Supplement S8*). A table outlining feasibility metrics and timing of assessment is available in Supplementary data online, *Supplement S9*.

### Exploratory primary outcome

There was no evidence to suggest a difference in the composite exploratory primary safety outcome between exercise and usual care groups (*P* = .99). One participant in the exercise group experienced exercise-induced syncope after developing ventricular standstill during exertion and one participant in the usual care group developed an episode of sustained VT. Both individuals are in good health without further complications. There was no evidence to suggest a between group difference in the burden of NSVT at 12 weeks (*P* = .99). During the study period, six (15%) patients in the exercise group and four (10%) patients in the usual care group underwent ICD implantation due to perceived increase of their arrhythmic risk (*P* = .737). See Supplementary data online, *Supplement S10* for changes in ESC risk score variables in all individuals who underwent ICD implantation. In all patients awaiting a primary prevention ICD, these were implanted following completion of the study.

### Exploratory secondary outcomes

**Figure 2:**
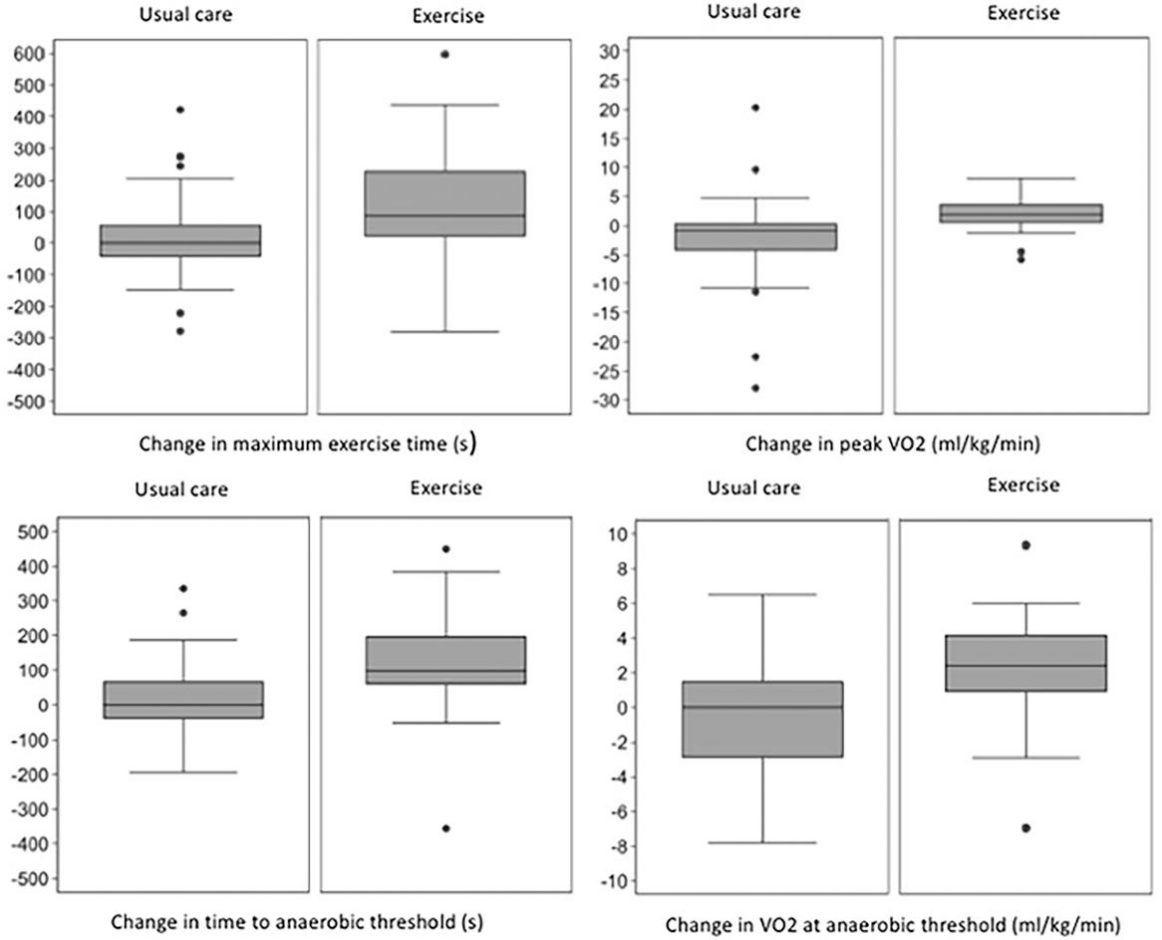
Change in exercise capacity from baseline to 12 weeks. Box whisker plots depicting raw median, IQR, and range data (clockwise from top left) for the change in maximum exercise time, peak VO_2_, VO_2_ at the anaerobic threshold, and time to anaerobic threshold

At 12 weeks, the exercise group demonstrated a greater increase in hours of PA engagement compared to the usual care group (2 ± 1.6 vs. 0.3 ± 2.3 h) (data not shown). The exercise time during cardiopulmonary testing increased by 121.4 s in the exercise group compared to 19.6 s in the usual care group, with a greater mean increase for the exercise group of 101.8 s (95% CI −21.1, 182.6). The mean change in peak VO_2_ was +1.9 mL/kg/min for the exercise group and −2.1 mL/kg/min for the usual care group with a mean between group difference of +4.1 mL/kg/min (95% CI 1.1, 7.1). The exercise group showed a greater mean increase of the time to AT [120.9 s vs. 13.8 s, between group difference +107.0 s (95% CI 42.5, 171.6)] and a greater VO_2_ at AT [2.0 mL/min/kg vs. −0.3 mL/kg/min, between group difference +2.3 mL/kg/min (95% CI 0.4, 4.1)] (*Figure 2*, *Table 2*).

**Table 2:** Within group post (T12)–pre (T0) data summaries and exploratory analyses of the difference in these changes between the two groups

	Exercise within group post–pre change			Usual care within group post–pre change			Per-protocol: non-parametric paired test (participants who completed trial)	Per-protocol: parametric unpaired *t*-test (ignoring potential departures from normality assumptions)		Intention-to-treat: permutation tests (all randomized participants)
	Mean/SD	Median (Q1–Q3)	*n*	Mean/SD	Median (Q1–Q3)	*n*	*P*-values	Estimated mean difference and 95% CI	*P*-values	*P*-values
Cardiovascular risk parameters										
BMI (kg/m^2^)	−0.7 (0.8)	−0.5 (−1, −0.2)	33	0.1 (0.7)	0 (0, 0.4)	34	<.001*	−0.8 (−1.1, −0.4)	<.001	<.001
SBP (mmHg)	−10.3 (8.9)	−10 (−16, −2)	34	−2.6 (9.9)	0 (−6, 0)	34	<.001*	−7.3 (−11.7, −2.8)	.001	.004
DBP (mmHg)	−1.8 (9.9)	0 (−10, 0)	34	−0.3 (8.1)	0 (−5, 0)	34	.357	−1.5 (−5.8, −2.9)	.505	.441
HbA1c (mmol/mol)	−0.4 (3.5)	0 (−1, 1.5)	32	0.1 (1.9)	0 (−1, 1)	30	.757*	−0.1 (−0.9, 1.1)	.460	.565
Cholesterol (mmol/L)	−0.2 (0.6)	−0.2 (−0.5, 0.2)	33	0.1 (0.7)	0 (−0.3, 0.4)	30	.247*	−0.3 (−0.6, 0.03)	.154	.700
HDL (mmol/L)	−0.01 (0.2)	−0.03 (−0.1, 0.08)	33	−0.1 (0.2)	−0.1 (−0.2, 0.06)	30	.615	0.1 (−0.04, 0.1)	.326	.367
LDL (mmol/L)	−0.2 (0.5)		32	−0.003 (0.5)		29	.086	−0.2 (−0.5, 0.1)	.167	.418
Cardiac biomarkers										
NT-proBNP (ng/L)	49.7 (193.7)	9 (−7, 48)	33	−40.1 (180.5)	−2 (−29, 25)	29	.287*	140.7 (62.7, 219.0)	.063	.137
Troponin (ng/L)	1.4 (4.2)	2 (−1, 3))	31	−2.9 (13.5)	0 (−3, 0)	29	.007*	3.0 (0.3, 5.6)	.096	.018
CPET parameters										
tAT (s)	120.9 (145.6)	97 (59, 197)	33	13.8 (114.8)	0 (−40, 67)	33	<.001*	107.0 (42.5, 171.6)	.002	.002
tMax (s)	121.4 (178.3)	88 (21. 228)	33	19.6 (148.8)	0 (−43, 58)	33	.002	101.8 (−21.1, 182.6)	.014	.016
VO_2_/kgAT (mL/kg/min)	2.0 (3.9)	2.4 (0.9, 4.2)	33	−0.3 (3.6)	0 (−2.9, 1.5)	33	.011*	2.3 (0.4, 4.1)	.019	.038
pVO_2_/kg (mL/kg/min)	1.9 (2.9)	1.9 (0.5, 3.6)	33	−2.1 (8.1)	−0.9 (−4.3. 0.4)	33	<.001*	4.1 (1.1, 7.1)	.009	.002
VE/VCO_2_	1.0 (2.6)	0.5 (−0.5, 1.7)	33	−0.4 (4.2)	0 (−1.9, 1.8)	33	.293*	1.4 (−0.3, 3.2)	.106	.395
Echocardiographic parameters										
LA volume (mL)	0.1 (24.2)	1 (−18, 16)	31	−3.9 (20.7)	−5 (−21, 11)	31	.477	2.9 (−7.8, 13.7.)	.487	.580
LVEDD (mm)	0.8 (3.1)	1 (−1, 3)	34	−1.4 (9.1)	0.5 (−2, 2)	34	.462*	2.2 (−1.1, 5.4)	.192	.555
LVWT (mm)	−0.1 (1.9)	0 (0, 1)	34	0.8 (2.7)	0 (−1, 2)	33	.346*	−0.9 (−2.1, 0.2)	.088	.567
E/A	0.01 (0.4)	0.7 (−.03, 0.3)	33	−0.03 (0.5)	0 (−0.3, 0.3)	32	.783	0.04 (−0.2, 0.3)	.685	.712
Average E/E′	−0.2 (1.6)	−0.4 (−1.2, −.6)	33	−0.5 (2.0)	−0.4 (−1.2, 0.7)	32	.595	0.4 (−0.5, 1.3)	.407	.555
Psychological and QoL measures										
HADSD score	−1.8 (2.8)	−1 (−3, 0)	33	−0.1 (1.8)	0 (0, 0)	33	.005*	−1.7 (−2.9, −0.5)	.005	.036
HADSA score	−2.7 (3.2)	−2 (−4, −1)	33	0.3 (2.1)	0 (0, 1)	33	*P* < .001*	−3 (−4.3, −1.7)	<.001	<.001
WHO-DAS II score	−3.6 (14.7)	0 (−4, 0)	33	0.2 (3.5)	0 (0, 0)	33	.297*	−3.8 (−9.1, 1.4)	.151	.422
SF-36 score	2.7 (18.4)		31	3.8 (9.1)		33	.191*	−1.08 (−8.3, 6.1)	.765	.169

^*^Indicates that there was some evidence for departure from the normality assumption in at least one group according to Shapiro–Wilk test. Hence, we presented both parametric and non-parametric per-protocol (PP) analyses for a quantitative insight into differences between the changes between groups. The intention-to-treat (ITT) analyses (based permutation tests) include all the randomized participants. All three types of *P*-values are consistent in their qualitative messages, and we present the estimated differences in the changes between groups and their 95% CIs. Troponin presents an exception due to a strong departure from the normality assumption which results in qualitative disagreement between the *t*-test (*P* = .096) and its non-parametric counterpart (*P* = .007). The non-parametric test is less powerful than the *t*-test but both PP non-parametric test and ITT analyses indicate some differences between the changes in the two groups as the *t*-test analyses violate the normality assumptions. This can be seen from the data summary as the mean is very different from the median and also from the location of the data as indicated by the median (Q1–Q3).

At 12 weeks, the exercise group showed a greater reduction in BMI and SBP (*Table 2*). There was no between group difference in cholesterol, HbA1c, cardiac biomarkers or any echocardiographic measures of cardiac morphology or function or ventricular ectopic burden (*Table 2*). Among the outcomes examining effects of exercise training on QoL, there were no differences between the exercise group and the usual care group at 12 weeks in SF-36 score and the WHO-DAS II score. Differences in favour of the exercise group were noted in the anxiety [HADSA −3 (95% CI −4.3, −1.7)] and depression [HADSD −1.7 (95% CI −2.9, −0.5)] scores (*Table 2*).

### Exercise group 6-month follow-up

**Figure 3:**
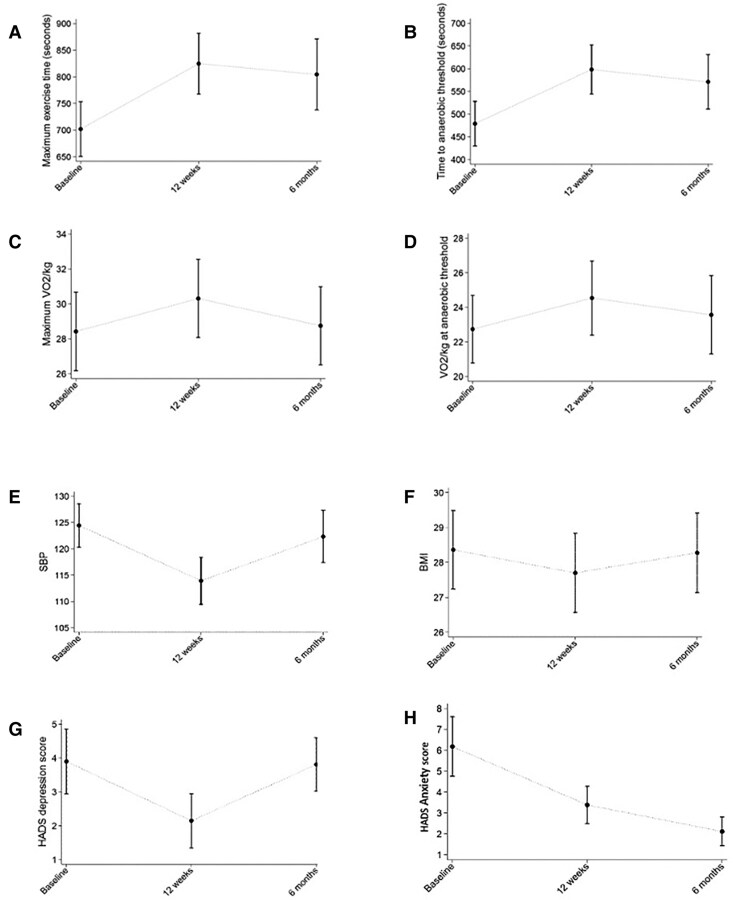
Change in the means and confidence intervals in (*A*) maximum exercise time ET, (*B*) time to anaerobic threshold, (*C*) VO_2_/kg at anaerobic threshold, (*D*) HADS anxiety score, (*E*) BMI, (*F*) systolic BP, (*G*) peak VO_2_/kg, (*H*) HADS depression score as estimated by the mixed models applied to the exercise group followed beyond the duration of the trial

At 6 months, self-reported PA levels suggested a return to usual baseline activity. Most observed improvements tended towards baseline values (*Figure 3*). This was supported by a significant effect of a quadratic term in time when a mixed model was fit to the data, for which the descriptive summary statistics for each three occasions (baseline, T12, and T6m) are presented in Supplementary data online, *Supplement S11*. No arrhythmic events or increased prevalence of NSVT was recorded.

## Discussion

Implementation of primary and secondary prevention strategies has resulted in expansion of the HCM population. As a result, there are greater numbers of younger, low-risk individuals, with mortality rates comparable to the general population.^31,32^ This underscores the need to evaluate high intensity exercise regimes that are likely to be attractive to younger individuals and reduce the long-term sequelae of a sedentary lifestyle. In this randomized, controlled feasibility study, we demonstrated that a supervised high intensity exercise programme was feasible in patients with HCM. Our results suggest potential gains in cardiorespiratory fitness, improvements in cardiovascular risk factors, and positive psychological outcomes. Importantly, there was no signal of increased arrhythmias (*Structured Graphical Abstract*).

### Feasibility

Thirty-four (85%) participants completed the 12-week exercise programme. This is similar to the UK national average (77%) for completion of a cardiac rehabilitation programme,^33^ and the completion rate reported by Saberi *et al*.^15^ for a home-based moderate intensity exercise programme in HCM. All individuals who completed the exercise programme adhered to a minimum attendance of 75% of exercise sessions, a value greater than that observed in cardiac rehabilitation studies (68%).^34^ Most participants (65%) achieved the upper limit of high intensity exercise (maximum HRR of 85%). The current study was feasible with respect to resources including equipment and the intervention, although staffing levels were higher to ensure safety.

### Exploratory outcomes

#### Safety

Our study suggests no difference in the exploratory composite safety outcome between exercise and usual care groups and no difference in the burden of NSVT at baseline and 12 weeks. These findings align with a small study exercising individuals at very high intensity (90%–95% of maximum HR) (*n* = 7) vs. moderate intensity (*n* = 8) which demonstrated improvements in peak VO_2_ without an increase in the incidence of serious arrhythmias in the former group.^35^ The results from both studies should, however, be interpreted with caution as neither was powered for the safety.

Ten (13%) patients were implanted with an ICD over the period of 12 weeks. In seven cases, the presence of new or repeat episodes of NSVT was the primary reason. Although the similar implantation rates between the exercise and usual care groups offer reassurance, our findings highlight the challenges of prolonged ECG monitoring which is becoming widely available. Prolonged ECG monitoring is likely to detect more episodes of NSVT compared with the annual 24–48 h ECG monitoring incorporated in the ESC HCM-SCD risk calculator. A study utilizing 14-day ECG monitors detected NSVT in 75% of patients, with only 23% and 45% of NSVT captured within the first 24 and 48 h, respectively.^36^ Therefore, the use of intensive monitoring may serve to increase the perceived risk, rather than the true risk of SCD. Supplementary data online, *Supplement S10* outlines in detail the change in ESC risk score variables in all individuals who underwent ICD implantation. Moreover, six patients who underwent primary prevention ICD implantation had a calculated ESC risk score of between 4% and 6% (an ICD may be considered), and the decision to implant an ICD was made in the context of a multidisciplinary meeting and shared decision making after extensive discussion with the patients. It is possible, therefore, that these patients may not have received an ICD in other centres.

#### Exercise capacity

Our findings indicate that regular, high intensity exercise may improve exercise capacity in patients with HCM. Patients in the exercise group increased their exercise time during CPET by 101.8 s and their peak VO_2_ by 4.1 mL/kg/min, compared to the usual care group. Moreover, the exercise group showed improved aerobic capacity, as evidenced by greater time to AT (+107.0 s) and VO_2_ at AT (+2.3 mL/kg/min) compared to the usual care group. There were no confounding effects of submaximal exercise testing given that the respiratory exchange ratio exceeded 1.1 in more than 95% of individuals in both groups at T0 and T12. Additionally, analysis of CPET data was blinded. The difference between groups remained when comparing peak VO_2_ (mL/min) (data not shown), suggesting that this was not driven solely by a reduction in BMI. Although the absolute increase of peak VO_2_ in the exercise group was modest (+1.9 mL/kg/min), these findings are in keeping with studies of home-based moderate intensity exercise programmes.^15^ Modest increases in peak VO_2_ may confer benefit. Studies in HCM population have demonstrated that lower peak VO_2_ predicts higher rates of mortality and morbidity^37,38^ and there may be as much as a 21% risk reduction in death or transplant for each 1 mL/min/kg increase in peak VO_2_.^39^

#### Cardiovascular risk factors

We showed a clinically meaningful reduction in cardiovascular risk factors, namely BMI (0.8 kg/m^2^) and SBP (−7.3 mmHg). A reduction of SBP by 5 mmHg may lead to a 9% reduction in CAD, 14% reduction in cerebrovascular accident, and 7% reduction in overall mortality.^40^ Furthermore, in a cohort of 425 patients with HCM, hypertension was present in 58% of black patients and 32% of white patients and was an independent risk factor for a composite outcome of cardiovascular death, cardiac arrest, or appropriate ICD therapy.^7^

#### Psychological and health status outcomes

There was no improvement in QoL scores. This may be due to the fact that both the SF-36 and WHO-DAS II are generic QoL/health status questionnaires which assess a range of general domains, of which physical functioning is a small part. Disease-specific questionnaires, such as the HCM symptom questionnaire (HCMSQ),^41^ are more likely to capture improvements in symptoms and physical functioning, the domain most likely to demonstrate improvement following an exercise programme. In fact, physical functioning was the only domain of the SF-36 questionnaire demonstrating a significant difference in those enrolled in a 16-week moderate intensity exercise programme compared to controls.^15^ However, the HCMSQ was validated following completion of the study.^41^ Its use may have demonstrated differences in QoL not observed with more generic assessment methods. However, we observed clinically meaningful reduction in both the anxiety (HADSA: −3) and depression (HADSD: −1.7) scores in the exercise group, as indicated by a change in score of 1.5 and 0.5, respectively.^42^

### Cardiovascular remodelling

At 12 weeks, there were no differences between groups with regard to cardiovascular remodelling, although it is unlikely that positive or adverse remodelling would occur within this timeframe. This is in keeping with studies in HCM patients who did not demonstrate any difference in LVWT, cavity size, or left ventricular diastolic dysfunction across PA levels,^43^ and the findings in individuals who participated in a 16-week moderate intensity exercise programme.^15^ Although studies in athletes with HCM indicate enhanced diastolic function compared to sedentary individuals,^13^ this is likely to depend on disease phenotype and the duration, frequency, and intensity of exercise, but may also reflect that those with milder disease phenotypes are more likely to take part in PA and participate in recreational or competitive sport. Although raised levels of troponin^44^ are known to be associated with adverse outcomes, there are no data regarding their prognostic significance in the context of HCM and exercise. In order to quantify a clinically relevant increase in cardiac biomarkers, larger studies with longer-term follow-up are required.

### Adherence to exercise

Individuals who actively engage in longer-term exercise programmes are more likely to maintain healthy levels of PA and sustain the benefits of exercise training. Our findings were consistent with studies comparing short to longer-term engagement in exercise, in terms of reduction in PA levels and loss of most observed benefits at 6 months.^45,46^ A potential solution to improve adherence to exercise may be through remote programmes^47,48^ given that moderate intensity exercise programmes have suggested that an unsupervised approach may be safe.^15^ Although such efforts may be hampered by safety concerns in HCM patients when exercising more vigorously, remote monitoring through the use of telehealth has great potential.^49^ Given that hard safety data in high intensity exercise in HCM are lacking, a hybrid approach may be preferable.

### Limitations

This study was subject to potential sampling bias as 290 individuals from the initial cohort (*n* = 636) were excluded and 125 declined participation. The majority of participants were male (84%) and white (63%), which limits extrapolation of the study findings to other patient groups. Furthermore, most participants did not exhibit a severe HCM phenotype, including significant left ventricular outflow tract obstruction, and the results of this study cannot be directly applied in instances of more advanced pathology. Participants were not blinded, and therefore, responses to psychological assessments may have been biased by their own expectations of participation. There was no difference in the composite primary outcome between the exercise and usual care groups, however, this was a feasibility study in a small cohort of individuals and therefore, was not powered for safety. Future studies might employ a cross-over/waiting list trial design in order to increase the numbers of participants in the exercise arm. Adverse events did occur, albeit in both groups, highlighting the need to monitor individuals and ensure appropriate follow-up to mitigate risk.

## Conclusions

A high intensity programme with individualized exercise prescription is feasible in patients with HCM, with potential gains in cardiorespiratory fitness, reduction in cardiovascular risk factors, and improvement of psychological measures. A large-scale study, in cohorts with greater heterogeneity, is required to corroborate findings and assess long-term safety of high intensity exercise in HCM.

## Supplementary Material

ehae919_Supplementary_Data

## Declarations

### Data Availability

The data underlying this article cannot be shared publicly due to the privacy of individuals that participated in the study. The data will be shared on reasonable request to the corresponding author.

### Pre-registered Clinical Trial Number

The pre-registered clinical trial number is ClinicalTrials.gov NCT05459467.
