# Supplementary material for: High intensity exercise programme in patients with hypertrophic cardiomyopathy: a randomized trial
Source: Eur Heart J. 2025 Mar 3;46(19):1803–15. doi: 10.1093/eurheartj/ehae919 (PMC12075935; doi:10.1093/eurheartj/ehae919)
Supplement: ehae919_Supplementary_Data [file ehae919_supplementary_data.pdf]

SAFE-HCM

This research project is funded by the charitable organisation Cardiac Risk in the Young

Cardiac Risk in the Young

Meet the team

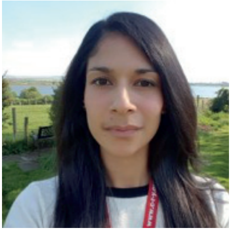

Dr Joyee Basu
Cardiology registrar & CRY Clinical Research Fellow

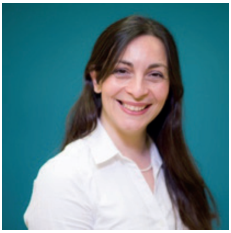

Dr Dimitra Nikoletou
Associate Professor Rehabilitation Sciences

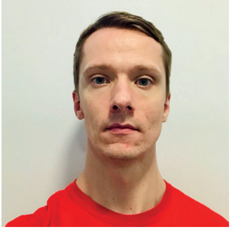

Shane Purcell
Cardiac Rehabilitation Exercise Instructor

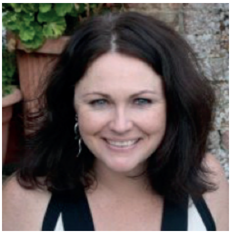

Carmen Lech
Senior Research Physiotherapist

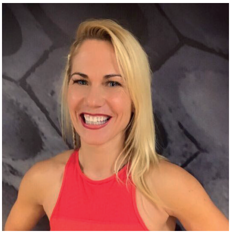

Paula Beck Simmonds
Cardiac Rehabilitation Instructor, personal trainer, fitness & rehabilitation coach at PB Fitness Training

PB Fitness Training
Reach your Personal Best

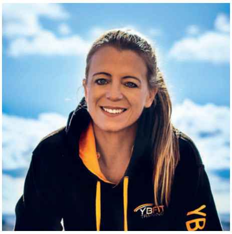

Claire Baker
Director of Fitness at training provider YBFIT Training & personal trainer

YBFIT
TRAINING

Contents

The heart 4

Hypertrophic cardiomyopathy 6

Home-based exercise 13

Lifestyle 22

Diary 28

The heart

Structure

The heart is made up of two chambers at the top (**atria**) and two chambers at the bottom (**ventricles**). The atria and ventricles are separated by a structure called the septum (**Figure 1**).

The heart has three layers, the endocardium, myocardium and pericardium (**Figure 2**). The endocardium is the innermost layer of the heart. The myocardium is the thick muscle layer of the heart that pumps the blood around. This is the layer that is affected by cardiomyopathies. The pericardium is made up of two layers and surrounds the heart. Pericardial fluid is contained within these layers which helps lubricate the layers as the heart pumps.

Blood flow

The blood flows from the body into the right atrium, through the tricuspid valve and into the right ventricle. The blood is pumped by the right ventricle into the lungs. From the lungs the blood travels through the pulmonary arteries into the left atrium, through the mitral valve and into the left ventricle. This blood is then pumped by the left ventricle into the aorta and then passes around the body (**Figure 3**).

Electrical activity

Each heart beat starts with an electrical impulse generated at the sinoatrial node (SAN). The electrical activity causes the atria to contract, allowing blood to pass through to the ventricles. The electrical activity then passes from the atria to the atrio-ventricular node (AVN) and into the ventricles causing them to contract. This allows the blood to pass to the lungs (from the right ventricle) and into the body (from the left ventricle) (**Figure 4**).

Figure 1: structure of the heart

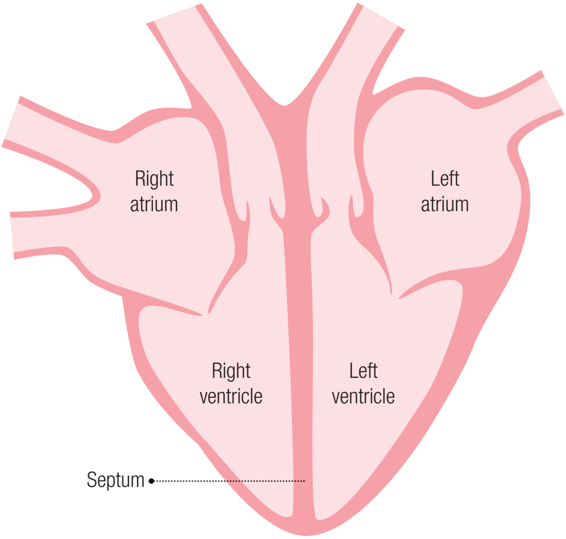

Figure 2: layers of the heart wall

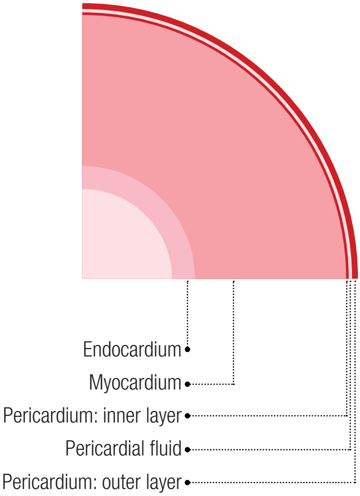

Figure 3: blood flow through the heart

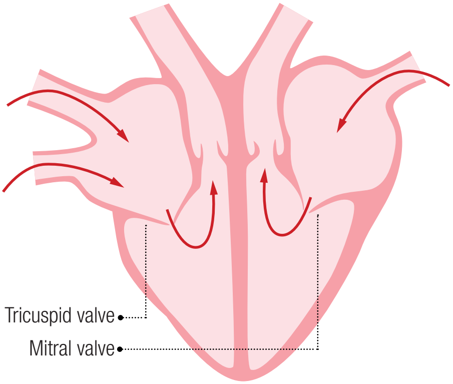

Figure 4: electrical activity in the heart

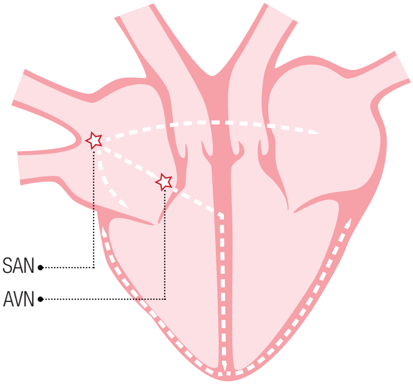

Hypertrophic cardiomyopathy

Hypertrophic cardiomyopathy (HCM) affects one in 500 people in the UK. It usually develops after puberty.

Inheritance

HCM is a genetic condition caused by a mutation (change) in a gene. Genes are made up of DNA. The order of the DNA within a particular gene provides instructions for cells to develop in a certain way. Genes are carried on chromosomes. There are 23 pairs of chromosomes where one chromosome in each pair comes from each parent (**Figure 5**).

HCM is usually caused by a mistake in the code of the DNA in one of the genes that we know is important for heart muscle cell development. It is inherited such that **an affected individual has a one in two chance of passing on the gene.** This is known as autosomal dominant inheritance. As it is a genetic condition multiple members of the same family may be affected. This is why it is important that all first degree relatives (mother, father, brother, sister, child) of an affected individual are screened for the condition with an electrocardiogram (ECG) and jelly scan (echocardiogram). The usual recommendation for **screening relatives** of an affected individual is annually from the age of 10 until 21 and five-yearly thereafter.

If a gene has been identified in an affected individual then other members of the family may also be able to have a genetic test. If family members are not found to have the gene then they will not be at risk of developing HCM and will not need further follow up. If this has not been offered to you please speak to your cardiologist.

Figure 5: chromosomes

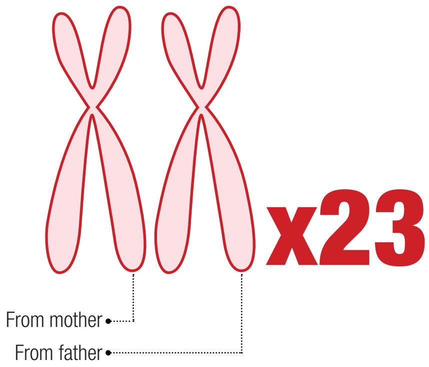

Hypertrophy

HCM causes thickening of the muscle in the left ventricle. Where the thickening occurs and how much thickening occurs varies between individuals. At a cellular level, normally the muscle cells lie in straight lines but in HCM they become disorganised and the heart may also show evidence of scarring. The heart becomes stiff and therefore less effective at pumping blood around the body.

The **heart may thicken in different places.**

Asymmetrical septal hypertrophy

This causes thickening of the wall between the right and the left ventricle.

In some cases the thickening can cause obstruction to the blood flow from the left ventricle to the rest of the body. This occurs when the thickening is in a position where, when the mitral valve opens, it touches the septum (**Figure 6**). This is known as left ventricular outflow tract obstruction (LVOTO).

This can also lead to blood leaking backwards through the mitral valve. This is known as mitral regurgitation.

Figure 6: asymmetrical septal hypertrophy with LVOTO

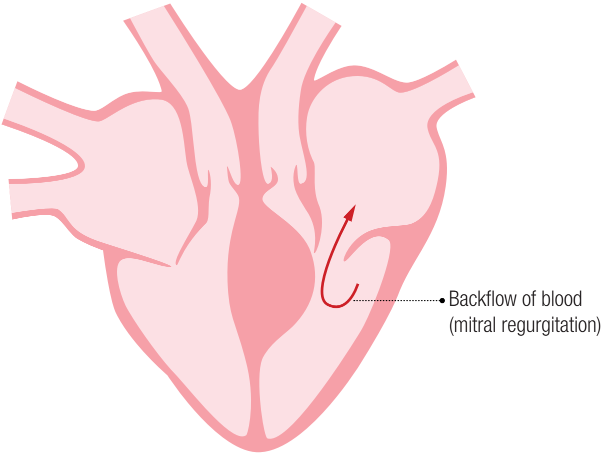

Symmetrical hypertrophy

The thickening of the heart is equally distributed (**Figure 7**).

Apical hypertrophy

The thickening occurs at the tip of the heart (the apex) (**Figure 8**).

Figure 7: symmetrical hypertrophy

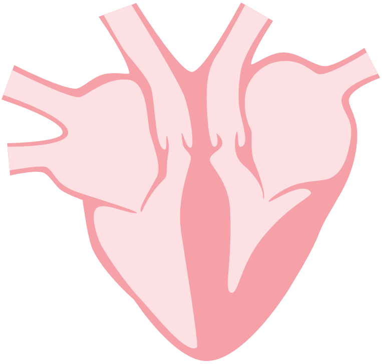

Figure 8: apical hypertrophy

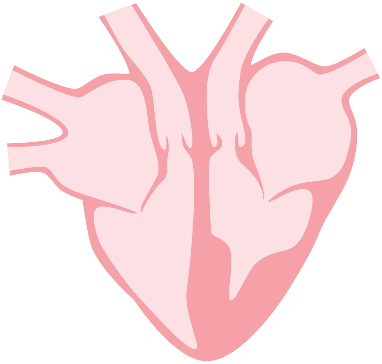

Diagnosis

Most people are diagnosed with HCM following screening due to an affected individual in the family, or incidentally. This may be due to the doctor noticing a heart murmur or the person undergoing tests for another reason and their ECG is found to be abnormal.

Some people present with symptoms including shortness of breath, chest pain, palpitations, which is the feeling of your heart beating, dizziness, and black outs.

If your doctor suspects a diagnosis of HCM they will usually refer you to a cardiologist with a specialist interest in inherited heart conditions for a series of further tests which includes:

Physical examination: the doctor will take your blood pressure, take your pulse and listen to your heart.

ECG: sticky electrodes will be placed on your arm, legs and chest. You will be connected via wires to an ECG machine. This is used to record the electrical activity in your heart.

Echocardiogram: this is a non invasive test where some jelly is applied to a probe which is placed on your chest. This emits ultrasound waves which reflect off different structures in the heart back to the probe at different speeds. This is used to build up a picture of your heart on a screen.

Exercise tests: this is similar to an ECG but you will be asked to exercise at the same time. This is usually performed

on a treadmill or a bike. If you undergo cardiopulmonary exercise testing (CPET) you may also be asked to breathe into special equipment at the same time to give additional information on how your body uses oxygen.

Holter monitoring: this is an ECG (only four to six leads rather than 12) which you wear for a specified amount of time, usually 24 to 48 hours. This is used to see whether you are having any heart rhythm disturbances (arrhythmias).

Magnetic resonance imaging (MRI): this requires you to lie in a tunnel. A small drip is put into your arm in order to be able to inject a dye that helps to look at scarring in the heart. The scanner uses a magnetic field and radio waves to build up a very detailed picture of your heart. This usually takes about an hour.

Complications

There are a number of important conditions to be aware of that occur as a direct result of HCM. People with HCM may suffer with arrhythmias. This is because the electrical activity can be affected by the areas of the heart which are scarred and the muscle cells are disorganised. The most common arrhythmia to originate from the ventricles is known as ventricular tachycardia. In this case the ventricles take over from the SAN in generating the heartbeat causing the heart to beat at a much faster rate. This is a dangerous arrhythmia as it can increase the risk of cardiac arrest which can lead to sudden death. Although most people with HCM live normal lives there is an increased risk of sudden death compared to the general population due to the risk of developing an arrhythmia.

As the ventricle is stiff this increases the pressure in the atria. This can lead to enlargement of the atria and can lead to abnormalities in the conduction of electrical activity through the atria. The most common arrhythmia to originate from the atria is known as atrial fibrillation. Atrial fibrillation causes the atria to beat fast and irregularly.

On the opposite end of the spectrum some people may develop heart block. This is where the electrical activity travels slowly, or may be blocked on the way to the ventricles. This causes a very slow heart beat and if it is too slow the person may need a pacemaker.

Treatment

There is no cure for HCM but the symptoms and complications can be managed with medicines and other interventions.

Medications

Medicines that may be offered to you are shown in **Table 1**.

Table 1: medications commonly used in the treatment of HCM

**Beta blockers** e.g. bisoprolol	
Slow the heart down which means the heart is working less hard and does not need as much oxygen. Can be used to treat LVOTO, arrhythmias and chest pain.	**Side effects:** tiredness, dizziness, cold hands and feet, erectile dysfunction.
**Calcium channel blockers** e.g. diltiazem, verapamil	
Slow the heart down which means the heart is working less hard and does not need as much oxygen. Can be used to treat LVOTO, arrhythmias and chest pain.	**Side effects:** dizziness, swollen ankles.
**Anti-arrhythmics** e.g. amiodarone	
Good at stabilising the heart and preventing atrial fibrillation and ventricular tachycardias. The most common anti-arrhythmic is amiodarone.	This can have side effects including sensitivity to sunlight (a strong sunscreen should be used), damage to the thyroid gland, liver and kidneys as well as the lungs. Therefore it is important to regularly monitor these organs with blood tests and a chest X ray.
**Anticoagulants** e.g. warfarin	
Atrial fibrillation can increase the risk of blood clots forming in the atria. If these clots form in the left hand side of the heart they can travel to the brain and cause a stroke. Anticoagulants thin the blood to reduce the risk of the clots forming.	**Side effects:** increased risk of bleeding.
**Diuretics** e.g. furosemide, spironolactone	
Help the body to lose water and therefore reduce the work that the heart has to do.	After starting diuretics it is important to have a regular check of your kidney function to show if too much or too little fluid is being removed from your body.<br>In people with diabetes diuretics may increase their blood sugar. In those with gout the condition may get worse.
**Disopyramide**	
An anti-arrhythmic drug, but is predominantly used to treat LVOTO.	**Side effects:** dry mouth, urinary retention.<br>A small number of people can develop arrhythmias.

Other treatments

Pacemaker: this can stop your heart from beating too slowly and is sometimes used to manage LVOTO. A pacemaker is implanted under local anaesthetic, a small box is placed under the skin just under the collar bone. It is connected to leads which are passed into the heart via a blood vessel (vein). Your heart rhythm is closely monitored and the pacemaker will only send an electrical impulse if your heart misses a beat.

Implantable cardioverter defibrillator: this has a pacemaker function but can also detect life threatening arrhythmias and deliver a small electrical shock to terminate them. This is inserted in a similar manner to a pacemaker. The difference is that the box is slightly larger and the procedure usually takes a little longer.

Alcohol septal ablation: this involves putting a small tube called a catheter up into the groin and feeding it up through a blood vessel (artery) to the heart. A small amount of alcohol is then injected into the branch of the heart artery that supplies the septum. This destroys some of the muscle in the septum causing the muscle to thin and reduces the obstruction (**Figure 9**). This can in some cases damage the electrical pathway and cause heart block which usually requires a pacemaker.

Surgical myomectomy: this is open heart surgery which involves removing a part of the thickened septum which relieves the obstruction. The risks of the surgery should be discussed with a cardiologist on an individual basis.

Heart transplantation: in suitable candidates a transplant may be considered.

Figure 9: alcohol septal ablation

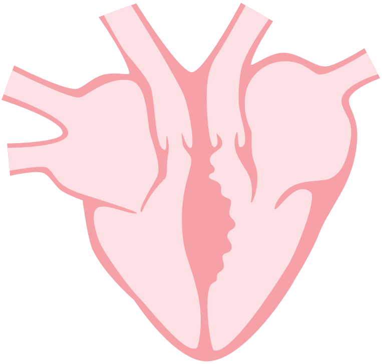

How can HCM affect you?

Exercise

Exercise is very important for health and well-being. You should discuss how much exercise you should do with a cardiologist. In general you should avoid stop start exercises such as football, squash and high intensity training as well as anything that causes you to strain such as heavy weights.

Weight

Weight loss helps to reduce the work of the heart and you should try to eat a healthy and balanced diet.

Smoking

Smoking can reduce the amount of oxygen carried in the blood due to the damage to the lungs which can put a greater strain on the heart to try and compensate for this.

Sex

Having sex may increase your heart rate and blood pressure, which can increase the amount of work your heart has to do and therefore give you symptoms. Therefore try to take things at a manageable pace. You may suffer with erectile dysfunction, this may be due to the medications you have been prescribed (beta blockers) or stress related to the diagnosis and/or living with the condition. You should use medication for erectile dysfunction only after consultation with your doctor, as it may worsen your symptoms or interact with other medications. It is important to note, if you have been prescribed Viagra or any other PDE5 inhibitor, you should not take this with anything containing nitrates as this can cause an extremely low blood pressure. These drugs should also be avoided for the same reason if you have LVOTO.

Pregnancy

This is usually safe but depends on the type of HCM you have and can cause increased strain on the heart. The medications you take may also have an effect on the baby. You should discuss pregnancy with a cardiologist who will be able to advise you accordingly.

Driving

The DVLA has clear guidelines on who should be restricted from driving. If you have no symptoms and are stable you should be able to drive but you should inform the DVLA of your condition. For more information visit www.gov.uk/health-conditions-and-driving, or call on 0300 790 6806.

Work

Depending on the type of job most people with HCM are able to continue working. Some jobs such as pilots and HGV drivers have strict guidelines on whether someone with HCM can continue to work. If your employer requires further information this can be provided by a cardiologist.

Travel, insurance & mortgages

You may be charged more for insurance, or have difficulty getting life insurance or a mortgage, if you are diagnosed with HCM. Contact Cardiac Risk in the Young (cry@c-r-y.org.uk) for information and advice.

Financial support

Some people with HCM may be eligible for Personal Independence Payment (PIP). This is a benefit which can help to pay for some of the costs that someone living with a chronic illness has.

Home-based exercise

Benefits of participating in a cardiac rehabilitation programme

- A **better understanding of how intensely to exercise**
- Improved physical **fitness**
- **Confidence** to go back to or start exercising
- Gain a healthier **body shape** and appearance
- Improved **strength and balance**
- Increased **energy** levels
- Increased **motivation**
- Improved **feel-good** factor
- Feeling more **relaxed**
- Improved **sleep** patterns
- Feeling **less isolated**.

Top tips to get the best out of your exercise programme

- **Avoid** physical activity **if you are unwell**
- **Keep hydrated** before, during and after your activity
- Wear the **right kit**, comfortable clothes and trainers
- **Build up** the pace of your activity **gradually,** make sure you slow down gradually (try not to stop suddenly)
- **Stop** exercising if you are in **pain**, feel **dizzy**, become **tired** or feel **unwell**
- **Stretch** after your activity to help avoid muscle stiffness
- **Contact your Trainers** if you have any questions on your programme.

How to keep motivated

- Do exercise that you enjoy, that way you are more likely to stick at it
- Add in extra activities that you can do with your friends and family e.g. swimming, walking, cycling etc.
- Remember the benefits of exercise, remind yourself that you will feel strong and more energized both physically and mentally
- Remember if you miss a day you can always exercise tomorrow, don't give up.

How to overcome barriers to exercise

- If you are feeling tired just remember once you start being more active you will have more energy in the long term
- Put exercise sessions into your diary so that it becomes part of your routine, treat it like you are making an appointment with yourself
- Remember any exercise is better then none
- Although this programme is free, there are plenty of ways to exercise for free such as walking or going for a run.
- If you are worried about getting injured, remember to build up exercise gradually and if you have any concerns contact your cardiac rehabilitation trainer for advice
- If you are worried about your weight, remember being active will help control your weight and we are here to support you
- If you are feeling embarrassed remember we are all in this together.

Home exercise plan

Don't forget to put your polar watch on.

	Level 1	Level 2	Level 3
**Warm-up**	• Shoulder roll<br>• Arm circles<br>• Side bends<br>• Heel raises<br>• High knees<br>• Walking lunges<br>• Side-stepping lunge<br>• Squats<br>• Pre-stretch: hamstrings; quadriceps; calves; chest; upper back. **Hold each stretch for up to 10 seconds.**	**Increase range of movement.**	**Increase range of movement.**
**Main: cardio**	**30 seconds' work, 30 seconds active recovery (feet moving).**<br>• March *or* jog on spot - x5<br>• Squat lunge<br>• Half jacks *or* full jacks<br>• Lunge back *or* kick and reach squat heel raises *or* squat jumps<br><br>**To finish:** march 30 seconds to 2 minutes to bring heart rate down.	**Increase pace and/ or try to make bigger movements.**<br>• Full jacks (instead of half)	**Increase to 40 to 50 seconds per exercise.**
**Muscle strength**	**30 seconds on each (aim for 6 to 8 repetitions).**<br>• Press-ups (box, three-quarter *or* full)<br>• Spider lunge<br>• Crunches *or* situps<br>• Oblique twists<br>• Triceps dips<br><br>**Remember:** have a few minutes of marching or slow movements to bring the heart rate down if necessary. Breathe evenly and drink plenty of water.	**30 seconds on each.**<br>• Three-quarter *or* full press-ups<br>• **Increase range*** of spider lunge, crunches *or* situps, and oblique twists<br>• Triceps dips: **use floor or chair to be higher off floor.**<br><br>* see videos	**40 to 50 seconds on each.**<br>**Increase speed and improve technique.**<br>• Full press-ups<br>• **Increase range*** of spider lunge, crunches *or* situps, and oblique twists<br>• Triceps dips: **extend legs or sit on something higher.**<br><br>* see videos
**Cool down**	**Hold for 15 to 30 seconds.**<br>• Shoulders, upper and lower back<br>• Hamstrings, quadriceps, adductors<br>• Obliques<br>• Chest	(As level 1)	(As level 1)

Exercise guide

Main (cardio) exercises

March

- Stand tall, feet hip width apart, chest up
- March legs up and down with your arms swinging lightly beside you.

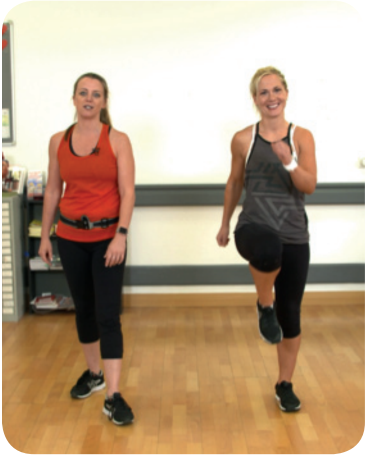

Squats & lunges

- Stand with feet hip width apart, chest up, hands on hips, keeping knees in line with the toes
- Bend knees to sit down into the legs
- Squeeze bottom as you rise up from the squat
- Step back, keeping feet hip width apart
- Bend the back knee so that both knees are at 90 degrees
- Come up and step forward back to feet hip width apart and repeat.

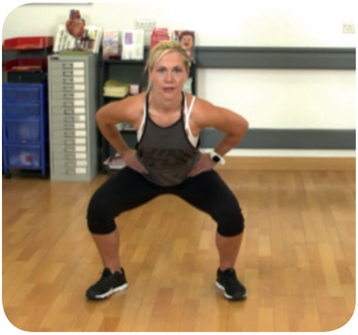

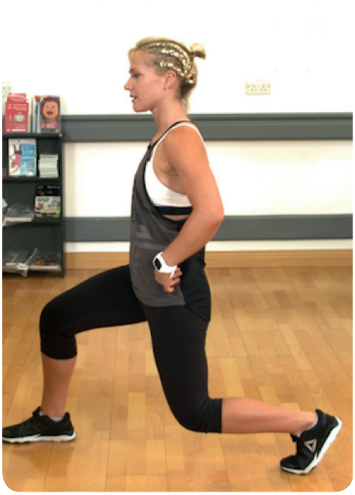

Half jacks

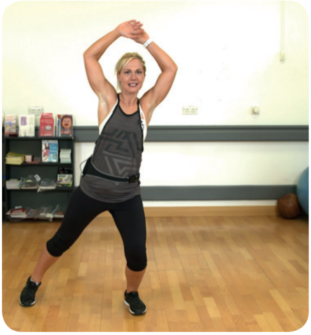

- Stand tall, feet hip width apart
- Step one leg out to the side laterally
- Take arms out wide to above your head
- Bring leg and both arms back in
- Repeat, changing legs each time.

Full jacks

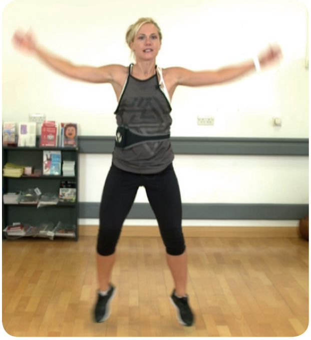

- As above but jump with both feet and take arms wide or up
- Land softly with feet flat to the floor, keeping knees soft.

Lunge back/ kick reach

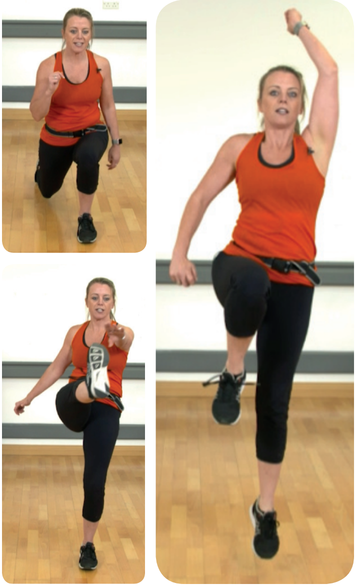

Level 1

- Stand with feet hip width apart, step back, bend back knee to a 90 degree angle keeping the front thigh parallel with floor
- Swing back leg up to waist height and reach with the opposite arm
- Keep chest up, draw in your stomach, keep hips in line.

Level 2

- As above with double pulse on back lunge.

Level 3

- As above and add hop at end of exercise.

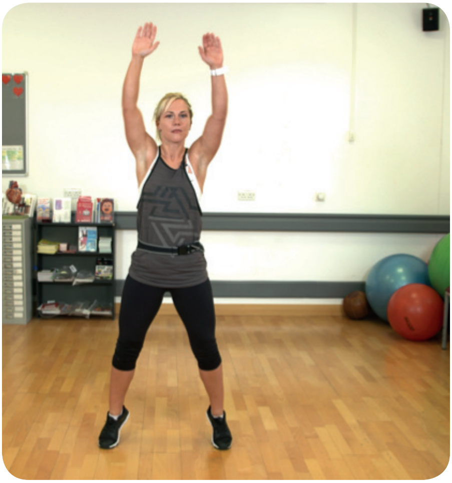

Squat heel raises

- Stand with feet hip width apart, chest up, keeping knees in line with the toes
- Bend knees to sit down into the legs
- Squeeze bottom as you rise up from the squat
- Lift heels as you come up and raise arms above your head
- Bring your heels and arms down and repeat.

Squat jumps

- As above but instead of lifting heels jump lifting knees
- Land lightly in the feet with heels down bending the knees and repeat.

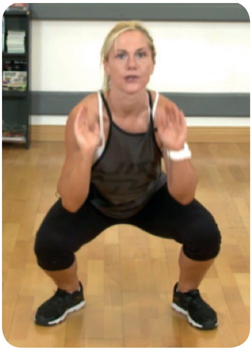

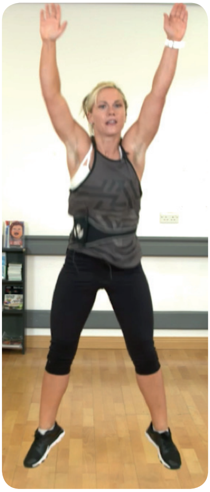

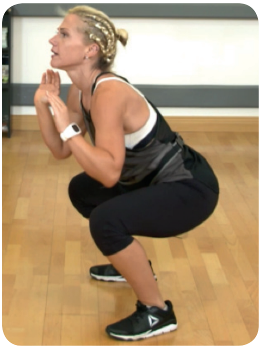

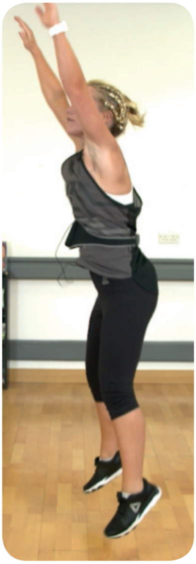

Muscle strength & conditioning

Press-ups

Level 1: box press-up

- On all fours, lower chest to elbow height and come back up.

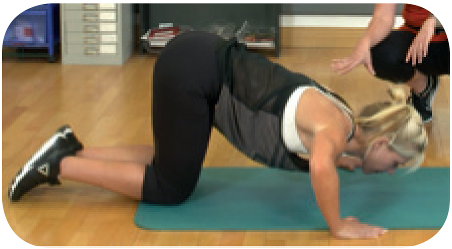

Level 2: three-quarter press up

- With hands widely spaced, keep knees hip width apart on the ground drawing your stomach towards your spine
- Keep bottom down in line with your back
- Lower chest to elbow height and come back up.

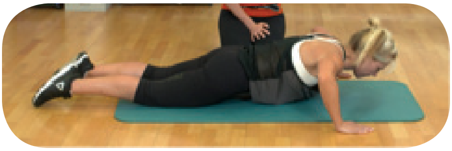

Level 3: full press-up

- As above, come up onto your feet so that your knees come off the mat.

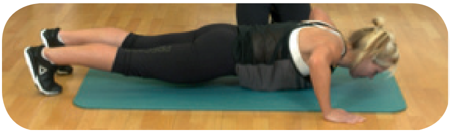

Spider lunge

Level 1

- Begin in plank position, contract your abdominal muscles
- Keep head in alignment with your back
- Bring one leg forward while keeping the rest of the body in the same position
- Repeat other side.

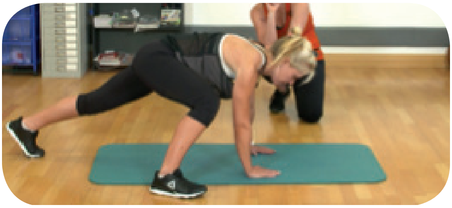

Level 2

- As above but bring leg up higher, nearer your shoulder.

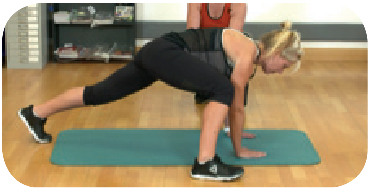

Level 3

- As above but bring your leg out to the side straight then up to your shoulder.

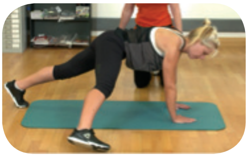

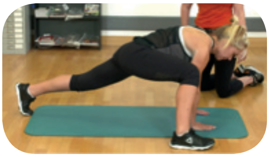

Abdominal curl

Level 1

- Lie on your back, knees bent, feet hip-width apart
- Draw your bellybutton towards your spine
- Tuck your chin in with your hands behind or beside your head
- Curl up peeling the shoulder blades off the floor
- Look forward or look through the gap between your knees
- Go back down to the floor and repeat.

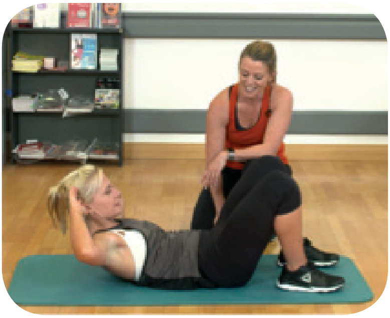

Level 2

- As above but lift legs so that you create a 90 degree angle at your knee.

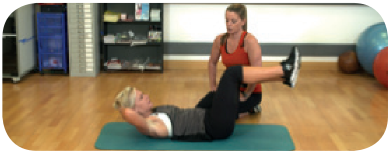

Level 3

- As above, again with knees bent, feet hip width apart, curl up whilst lifting arms up keeping them straight, beside your ears.

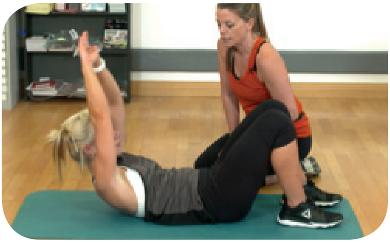

Oblique twists

Level 1

- Lie on your back, place hands next to head/ temples or softly behind the head
- Keep elbows out wide and lift elbow towards the opposite knee, twisting your torso.

Level 2

- As above but lift legs so that you create a 90 degree angle at your knees.

Level 3

- As above but bend opposite knee and extend other leg as you twist.

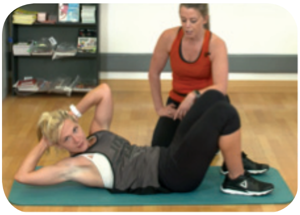

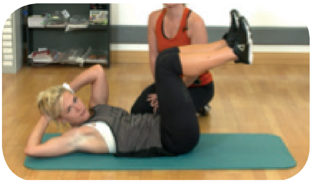

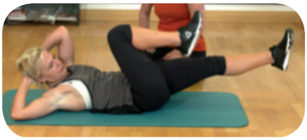

Triceps dips

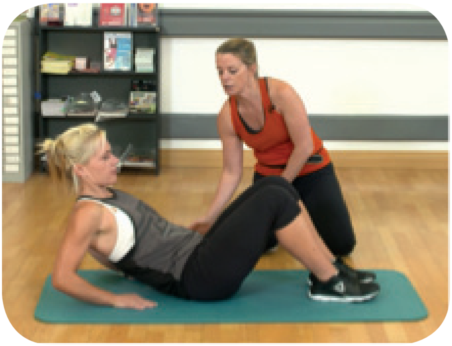

Level 1

- Start seated with knees bent and feet on floor, place hand behind you directly under shoulders with hands facing forwards
- Bend elbow, take upper body down towards the floor keeping your back straight back and push back up
- As you do this keep your elbows in alignment with your shoulders
- Keep looking forward throughout the exercise.

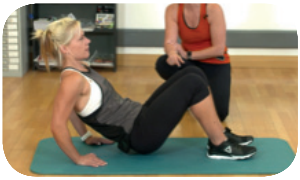

Level 2

- As above but with your bottom off the floor.

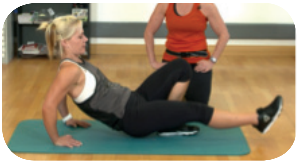

Level 3

- As above but with a leg extended out.

Please ensure you stretch out afterwards.

Links to SAFE-HCM YouTube videos

	Level 1	Level 2	Level 3
Warm up	GO		
Marching and jogging	GO	GO	GO
Squat lunge	GO	GO	GO
Jacks	GO	GO	GO
Lunge back & kick reach	GO	GO	GO
Sqaut heel raises & squat jumps	GO	GO	GO
Muscle strength	GO	GO	GO
Warm down	GO		

Lifestyle

Activity & exercise

Regular activity and exercise is important for everyone but even more important for people with cardiac conditions. The benefits include improvement in your general fitness, improvement in symptoms of breathlessness during activities such as walking uphill, and improvement in mood.

In this programme, your **individualised intensity of training will be set at the first session** and you will learn to monitor yourself. You will be able to progress in intensity and it is important that you follow these steps when you do your exercise at home. You can choose pleasurable activities and hobbies that encourage you to reach your set intensity or follow the set programme you do at the hospital.

You should be able to get out of breath but **excessive breathlessness or chest pain are symptoms to watch out for.** These may indicate the need to reduce the intensity or stop exercising. We will discuss tips to prevent getting these symptoms and how to manage them effectively if they occur.

Finally, it is important to consider not only your daily activities and how much exercise you perform but also **the amount of sitting hours during your average day.**

Occupational sitting & sedentary behaviour

It is now well known that **sitting for prolonged periods of time is harmful to health.** Even when people exercise, prolonged sitting can counterbalance the benefits of exercise. Occupational sitting is a term used to describe sedentary behaviour at work, for example when work involves sitting in front of a computer throughout the day. If your work involves prolonged sitting, then try the following tips:

Consider **changing your desk to a standing desk.** This is now an option for many employees and means you can alternate sitting to standing. You can still have an adjusted chair but you reduce the hours spent sitting down. Research has shown that people who use standing desks are more alert during the day and less tired when they get home.

If this option is not available, try to **stand and walk about regularly.** You could arrange an alarm on your mobile to remind you to take a walk or get up and stretch.

Take the stairs instead of the lift as many times as you can. If you are working on a high floor, think about getting out two floors below (or even above) and walking the remaining floors.

Think about a short **walk during your lunch break.** A 15 minute walk every lunchtime will accumulate benefits over a week.

If you are using the bus, **get off one or two stops before your stop** and walk the rest of the distance.

Try to **stand at every opportunity.**

Managing symptoms

The most common symptoms are chest pain and shortness of breath. You may have heard the term 'self-management' when it comes to symptoms. This refers to being educated about the signs that mean 'urgent action is required' and signs that mean 'you need to adjust the activity/ exercise, medication or behaviour'.

Box 1: Borg Rating of Perceived Exertion scale

6	No exertion at all.
7 to 8	Very, very light.
9 to 10	Very light.
11 to 12	Fairly light.
13 to 14	Somewhat hard.
15 to 16	Hard.
17 to 18	Very hard.
19 to 20	Very, very hard (maximal exertion).

© Gunnar Borg 1970, 1985, 1994, 1998

An example is breathlessness. When starting an exercise programme, especially if you have not exercised for a while, it is expected that you will experience a degree of breathlessness as your muscles, including your heart, start working harder. There are several methods to monitor whether your breathlessness is appropriate. The Borg Rating of Perceived Exertion scale (**Box 1**) has numbers that indicate the safe level of your perceived exertion, which corresponds to your ability to exercise and talk. For example, imagine you are walking uphill with a friend. The idea is that, if you can talk to your friend while walking uphill, even though you feel breathless this represents an appropriate intensity of exercise.

As the safe intensity of training is very individual in this group, we will discuss the numbers in the scale that correspond to your level.

Diet

Muscles need oxygen to work efficiently but they also need fuel. If you don't get some food into them you may feel dizzy or not get as much out of a session as you might otherwise. It's also important to keep hydrated, so keep drinking during exercise, and before, especially if you exercise first thing in the morning. The amount you need will vary from person to person, but it's about eight glasses of water a day, or two to three litres.

The picture below shows the **Eatwell guide, which is the current recommendation for a healthy mix of food.** This shows roughly a third of your food should be fruit and vegetables and a third should be starchy foods such as rice, pasta and potatoes. Note that portion size is also important – eating huge plates of food won't help even if it is precisely in these percentages!

It's also good to go for the **lower salt options** where possible. Eating slightly more meat, fish and beans may also be good, as they contain protein which helps to build muscle. These could replace some of the starch, but still keep a quarter of your food as pasta, potatoes etc.

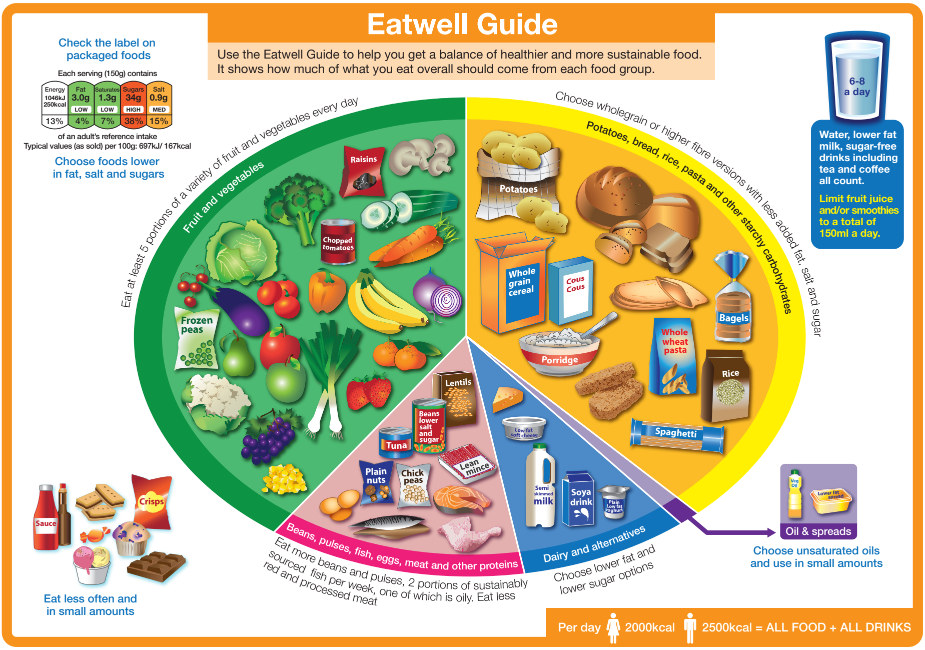

Source: Public Health England in association with the Welsh Government, Food Standards Scotland and the Food Standards Agency in Northern Ireland
© Crown copyright 2016

Other tips for a good diet

Eat at least **five portions of fruit and veg a day.**

Eat **two portions of fish a week.**

Try to eat **brown carbohydrates** (wholemeal bread, rice, pasta etc.) rather than white ones.

Limit the amount of refined carbohydrates (sugar and sweets) you eat. Small amounts are okay in moderation.

Vegetable fats are better than animal fats. Try to eat lean meat and, if you need to fry, use a small quantity of something like sunflower oil.

Although exercise burns up calories, **be careful not to overeat** after exercising.

Relaxation

What is relaxation?

Relaxation can be described as an **absence of stress, both physical and mental.** It is often referenced by the release of tension in muscles and the lengthening of muscle fibres, as opposed to muscle fibre shortening which accompanies muscle tension and contraction (Payne & Donaghy, 2010).

Relaxation can signify a general state of being, or as a technique to becoming more relaxed.

> *'[Relaxation is] a state of ease characterised by limited body tension and freedom from unnecessary worries and fears. It is associated with feelings of warmth and tranquillity…'*
> (Payne & Donaghy, 2010).

It is considered to be an interplay of both the physiological systems of the body (major organs, nerves and muscles), and the psychological system of the mind, feelings and emotions.

What is stress & how does it affect us?

Stress is a response to fear or anxiety governed by our autonomic nervous system. The autonomous nervous system is made up of two parts: the sympathetic nervous system and the parasympathetic nervous system.

The **sympathetic nervous system** responds to threat by releasing adrenaline into the blood stream. Another hormone called cortisol works to provide fuel for the 'fight or flight' response and stimulates the immune system.

The **parasympathetic nervous system** restores the body to calm once the threat or challenge has subsided. In states of chronic stress the body is less able to restore this system.

Prolonged stress, with long-term raised levels of cortisol, impairs the immune system, making it weaker. Stress also impairs quality of sleep. In turn, poor sleep puts more stress on the body.

An estimated 80% of modern disease originates in stress (Powell & Enright, 1990).

It is believed that having a significant illness contributes to being stressed. **Relexation can therefore be particularly beneficial for people living with a health condition.**

What are the benefits of relaxation?

- Improved health.
- Increased sense of well-being.
- Improved mood.

Where is the best place for me to practise relaxation?

- At home.
- In a garden or a park.
- When preparing for bed or before getting out of bed.
- When doing recreational activities e.g. sitting on a park bench or going for a walk.
- Anywhere when you have a moment.

When is the best time to relax?

- When preparing for bed or before getting out of bed.
- At a set time of the day which is yours to define.
- Anytime you find a moment.

Potential barriers to practising relaxation

- Finding the time.
- Having 'the space' – e.g. when others won't interrupt.
- Motivation.

Motivation

It is easy to put off relaxation and plan to do it later. However relaxation works best when done 'little and often' so it's best to practise it regularly.

It can be difficult to 'just do it' when you're on your own. Some people find it helpful to join groups, such as walking groups, or to set time aside for activities which they enjoy and have no deadlines, like hobbies.

Homework activity: *identify a time this week when you can practise relaxation. Practise the tense and relax technique (**Box 2**) for five minutes, noticing your breathing.*

Box 2: tense & relax technique for relaxation

A tense-release cycle involves: tensing the muscles in a part of the body; holding the muscles **tense for five seconds**; releasing the muscles so they **relax over 10 to 15 seconds.**

While practising the technique, **focus on the movements and sensations** of tensing and relaxing the muscles.

For example, for your right hand: begin by clenching your right hand… make a fist… make it tight... notice the sensation of tension in the hand and forearm while you hold it… then let go… feel the hand and forearm becoming relaxed and comfortable…warm and relaxed… relaxed and heavy…

First session: go through the sequence below. Hold each tense for five seconds, relax over 10 to 15 seconds, then start the next tense.

- Clench right hand, then relax
- Clench left hand, then relax
- Bend right elbow, then staighten by pressing your wrist into the arm of a chair
- Bend left elbow, then staighten by pressing your wrist into the arm of a chair
- Raise eyebrows and wrinkle forehead, then relax
- Bring eyebrows together and frown, then relax
- Screw eyes up tight, then relax
- Press tongue against roof of mouth, then relax
- Press lips together, then relax
- Press head against the back of a chair, then relax
- Press chin down onto collar bone, then relax
- Hunch shoulders up to ears, then relax
- Brace shoulders back to bring shoulder blades together, then relax

Second session: tense-release of chest, stomach, back, legs and feet. Go through the sequence below. Hold each tense for five seconds, relax over 10 to 15 seconds, then start the next tense.

- Tense the muscles that bring the stomach in, then relax
- Arch back so spine leaves the chair, then relax
- Tense buttocks by pressing feet down into the floor, then relax
- Raise heels with toes remaining on the floor, then relax
- Raise front part of the foot keeping heels on the ground, then relax

Diary

Complete the exercise diary using the Borg scale (**Table 2**) to assess the intensity of your activity.

Table 2: assessing your exercise effort using the Borg Rating of Perceived Exertion scale

How you might describe your exertion	Borg rating of exertion	Examples (for most adults under 65 years old)
None	6	Reading a book, watching TV.
Very, very light	7 to 8	Tying shoes.
Very light	9 to 10	Household chores such as folding clothes.
Fairly light	11 to 12	Walking through the supermarket or other activities that require some effort but not enough to speed up your breathing.
Somewhat hard	13 to 14	Brisk walking or other activities that require moderate effort and speed your heart rate and breathing but don't make you out of breath.
Hard	15 to 16	Cycling, swimming or other activities that take vigorous effort and get the heart pounding and make breathing very fast.
Very hard	17 to 18	The highest level of activity you can sustain.
Very, very hard	19 to 20	A finishing kick in a race or other burst of activity that you can't maintain for long.

© Gunnar Borg 1970, 1985, 1994, 1998

	Activity	No. minutes active	Activity intensity (Borg scale)
**Week 1**			
Day 1			
Day 2			
Day 3			
Day 4			
Day 5			
Day 6			
Day 7			

	Activity	No. minutes active	Activity intensity (Borg scale)
**Week 2**			
Day 1			
Day 2			
Day 3			
Day 4			
Day 5			
Day 6			
Day 7			

	Activity	No. minutes active	Activity intensity (Borg scale)
**Week 3**			
Day 1			
Day 2			
Day 3			
Day 4			
Day 5			
Day 6			
Day 7			

	Activity	No. minutes active	Activity intensity (Borg scale)
**Week 4**			
Day 1			
Day 2			
Day 3			
Day 4			
Day 5			
Day 6			
Day 7			

	Activity	No. minutes active	Activity intensity (Borg scale)
**Week 5**			
Day 1			
Day 2			
Day 3			
Day 4			
Day 5			
Day 6			
Day 7			

	Activity	No. minutes active	Activity intensity (Borg scale)
**Week 6**			
Day 1			
Day 2			
Day 3			
Day 4			
Day 5			
Day 6			
Day 7			

	Activity	No. minutes active	Activity intensity (Borg scale)
**Week 7**			
Day 1			
Day 2			
Day 3			
Day 4			
Day 5			
Day 6			
Day 7			

	Activity	No. minutes active	Activity intensity (Borg scale)
**Week 8**			
Day 1			
Day 2			
Day 3			
Day 4			
Day 5			
Day 6			
Day 7			

	Activity	No. minutes active	Activity intensity (Borg scale)
**Week 9**			
Day 1			
Day 2			
Day 3			
Day 4			
Day 5			
Day 6			
Day 7			

	Activity	No. minutes active	Activity intensity (Borg scale)
**Week 10**			
Day 1			
Day 2			
Day 3			
Day 4			
Day 5			
Day 6			
Day 7			

	Activity	No. minutes active	Activity intensity (Borg scale)
**Week 11**			
Day 1			
Day 2			
Day 3			
Day 4			
Day 5			
Day 6			
Day 7			

	Activity	No. minutes active	Activity intensity (Borg scale)
**Week 12**			
Day 1			
Day 2			
Day 3			
Day 4			
Day 5			
Day 6			
Day 7			

Notes

Cardiac Risk in the Young

Registered Charity No: 1050845

CRY is a non-profit UK charity established to:

- save young lives
- help those affected

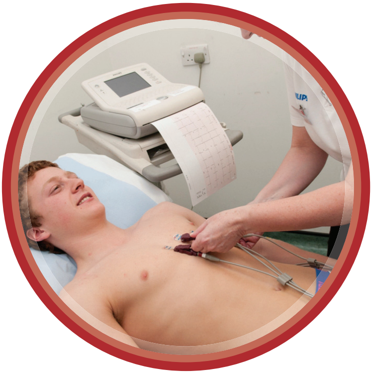

CRY is preventing young sudden cardiac deaths through awareness, support, screening and research.

Awareness
amongst medical practitioners and those at risk.

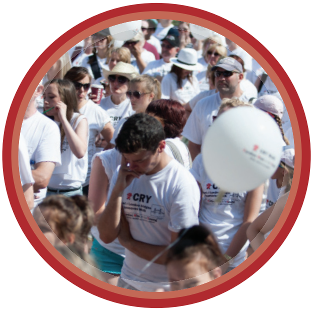

Support
Emotional and medical support after a young sudden death and for those living with an inherited heart condition.

Screening
offering young people the choice to be tested and identify cardiac conditions.

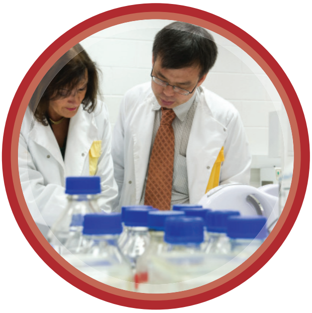

Research
to inform policy and practice.

CRY represents the thousands of families whose apparently fit and healthy children, partners and parents have died suddenly from undiagnosed cardiac conditions.

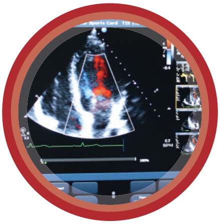

CRY believes the frequency of YSCD can be dramatically reduced by making heart screening available to all young people between the ages of 14 and 35.

www.c-r-y.org.uk **01737 363222** **Registered Charity No: 1050845**

CRYVideos **CardiacRiskintheYoung** **@CRY_UK**

First edition.
Copyright © *Cardiac Risk in the Young, 2018*
